# Incomplete Lineage Sorting and Hybridization Statistics for Large-Scale Retroposon Insertion Data

**DOI:** 10.1371/journal.pcbi.1004812

**Published:** 2016-03-11

**Authors:** Andrej Kuritzin, Tabea Kischka, Jürgen Schmitz, Gennady Churakov

**Affiliations:** 1 Department of System Analysis, Saint Petersburg State Institute of Technology, St. Petersburg, Russia; 2 Institute of Experimental Pathology (ZMBE), University of Münster, Münster, Germany; 3 Institute of Bioinformatics, Faculty of Medicine, University of Münster, Münster, Germany; 4 Institute of Evolution and Biodiversity, University of Münster, Münster, Germany; Cornell University, UNITED STATES

## Abstract

Ancient retroposon insertions can be used as virtually homoplasy-free markers to reconstruct the phylogenetic history of species. Inherited, orthologous insertions in related species offer reliable signals of a common origin of the given species. One prerequisite for such a phylogenetically informative insertion is that the inserted element was fixed in the ancestral population before speciation; if not, polymorphically inserted elements may lead to random distributions of presence/absence states during speciation and possibly to apparently conflicting reconstructions of their ancestry. Fortunately, such misleading fixed cases are relatively rare but nevertheless, need to be considered. Here, we present novel, comprehensive statistical models applicable for (1) analyzing any pattern of rare genomic changes, (2) testing and differentiating conflicting phylogenetic reconstructions based on rare genomic changes caused by incomplete lineage sorting or/and ancestral hybridization, and (3) differentiating between search strategies involving genome information from one or several lineages. When the new statistics are applied, in non-conflicting cases a minimum of three elements present in both of two species and absent in a third group are considered significant support (p<0.05) for the branching of the third from the other two, if all three of the given species are screened equally for genome or experimental data. Five elements are necessary for significant support (p<0.05) if a diagnostic locus derived from only one of three species is screened, and no conflicting markers are detected. Most potentially conflicting patterns can be evaluated for their significance and ancestral hybridization can be distinguished from incomplete lineage sorting by considering symmetric or asymmetric distribution of rare genomic changes among possible tree configurations. Additionally, we provide an R-application to make the new KKSC insertion significance test available for the scientific community at http://retrogenomics.uni-muenster.de:3838/KKSC_significance_test/.

## Author Summary

The presence/absence patterns of transposed elements, so called jumping genes, provide invaluable information about evolution. Unfortunately, there is still no clear all-encompassing analysis of the statistical significance of insertion patterns, and the single existing model of insertion data is no longer sufficient for the emerging genomic era. Here, we have provided a comprehensive statistical framework for testing the significance of support for phylogenetic hypotheses derived from genome-level presence/absence data such as retroposon insertions and for evaluating such data for different evolutionary scenarios, including polytomy, incomplete lineage sorting, and ancestral hybridization. This statistical framework is especially important for high-throughput applications of current and upcoming genome projects due to its treatment of unlimited numbers of testable markers, and is embedded in a user-friendly R-application available to the scientific community online. Finally, a reliable, adaptable calculation for the significance of support for phylogenetic trees derived from retroposon presence/absence data is now available.

This is a *PLOS Computational Biology* Methods paper.

## Introduction

In their pioneering work, Ryan and Dugaiczyk [1] first proposed using Short INterspersed Element (SINE) insertions as phylogenetic markers with the suggestion: “we submit that the chronology of divergence of primate lines of evolution can be correlated with the timing of insertion of new DNA repeats into the genomes of those primates”. Although their originally detected insertions were of no direct phylogenetic relevance, subsequent studies fostered this innovative idea, and systematically searched for retroposon insertions as genomic landmarks of phylogeny (e.g. [2],[3]).

While the current most popular use of DNA sequence comparisons to deduce phylogenetic relationships must make do with only four possible character states (ACGT), retroposon insertions can theoretically produce millions of different character states corresponding to the large number of random genomic insertion sites, and thereby requires special statistics to deal with such large numbers of character states. Important is, that the inserted element itself does not encode the character state, but rather the character state derives from the exact genomic position of the inserted element. The probabilities of two independent random insertions of the same element at the same genomic location in two unrelated lineages or the exact deletion of an orthologous element are negligible but not excludable (see also Discussion). For example, the probability of parallel SINE insertion in primates is calculated to be about 0.05% [4] and precise SINE excision to be less than 0.5% [5]. More importantly, inexact parallel insertions or deletions are easy recognizable by careful analysis of the complex structure of each individual diagnostic element insertion, enabling these loci to be excluded from further analysis. The character polarity of these markers is, in contrast to sequence data, unambiguous: presence indicates the derived state and absence the plesiomorphic condition (for additional information on the marker system see [6]). But it should also be mentioned, that presence/absence markers are, in contrast to sequence data, not universally available. Their accumulation is not clocklike, and therefore they are not suitable for calculating exact branch-length or population size. A synergistic application of both marker systems is the most efficient way to extract historical information from species.

An ideal phylogenetic marker evolves neutrally [7]. Unfortunately, such neutral or nearly neutral markers then tend to diverge beyond recognition in relatively short times and are therefore not suitable for deep phylogenetic comparisons. At the sequence analysis level, a compromise is to consider more conserved nucleotide positions (e.g., the second position of codons) taking into account that such positions are less neutral and therefore may lead to only a limited phylogenetic statement. On the other hand, slight natural selection rarely complicates phylogenetic analysis, as it usually involves only rate shifts, while “balancing selection” is a real challenge [8]. Retroposon insertions, by contrast, are unrestricted, random, almost exclusively neutral events, and therefore virtually free of any converging effects, fulfilling essentially the strict precondition of neutral evolution [9]. Due to the complex structure of inserted elements, retroposon insertions are recognizable for tens or hundreds of millions of years and are highly resistant to insertion saturation, hence resistant to post-insertional state changes. The degree of natural selection on retroposon insertions correlates with the region of insertion. Apart from the very rare cases of insertions into functionally significant structures (regulatory areas, intron boundaries, or coding sequences), the overwhelming majority of random integrations have no functional or selective importance. Any insertion, independent of where it takes place, is a unique event and post-insertional removal in a descendent lineage is easily recognizable by the highly complex traces that the insertion process leaves behind, enabling such markers to be omitted from further analysis. As explained before, mutations within an element do not compromise its phylogenetic value as a unique presence/absence marker. Diagnostic elements are extracted following strong criteria of orthology and only when they are clearly recognizable in all investigated lineages or when they can be irrefutably defined as absent are they used for phylogenetic analysis.

Another big advantage of this attractive marker system is its relative lack of conflicting data [6]. When such conflicts do arise, their origins are more easily recognized than those of simple sequence changes. One of the avoidable but still most common sources of apparently conflicting presence/absence patterns of retroposed elements is the violation of a strict definition of orthology. In most instances of mammalian retrotranspositions, the process of insertion generates specific target site duplications (TSD) of 8–30 nts flanking all inserted elements [10]. It is important to carefully compare the identity of such TSDs to the unoccupied site of distantly related reference species to clearly confirm the orthology of these loci. The consistent orientation of inserted elements and congruent element types in all analyzed species is another essential criterion for orthology. Furthermore, shared truncations of, or random indels in, elements can help to verify orthology after carefully considering potential hotspots of indels and breakpoints. In the most current investigations only loci with a clear signature of presence/absence in all investigated species (with sequence similarity >70%) are considered [3,11].

A second source of apparently conflicting presence/absence patterns in retrophylogenomics is incomplete lineage sorting during evolution, whereby polymorphic conditions of presence/absence states at the time of the formation of new species might lead to a random distribution of presence or absence states. Such character state polymorphism can similarly influence all types of polymorphic molecular or anatomical characters. Fixation starts with the appearance of an individual change in a population and continues until all individuals of the subsequent populations inherit the change, which can take several million years depending on effective population size [12] and is easily determined by *t* = 4*N*_e_ (where t = generations, multiplied by 25 years for humans will lead to the estimated real time and *N*_e_ is the expected ancestral effective population size, e.g, 20,000 for humans). Accordingly, for humans a fixation time of about 2 million years can be estimated. Corresponding to the neutral theory of molecular evolution, the fixation of a previously polymorphic marker depends on the size of the founder population (the smaller a population the sooner a neutral marker is fixed) and generation time (the shorter the generation time the sooner a marker is fixed). For primate populations 1–3 million years are usually sufficient to fix most markers [12,13]. Therefore, especially in rapid successive radiations and in young terminal branches, retroposed elements that entered part of a population may not yet have been uniformly fixed before the next step in speciation occurred. In most such cases, this incomplete lineage sorting leads to a random presence or absence state of markers in lineages and, due to the relative unambiguity of retroposon insertions (presence or absence) and their insertion complexity, is more easily recognized as an equal or symmetric polytomy (all three possible topologies of three related species are more or less equally supported) [14] than a simple sequence change. For example, the highly debated phylogenetic relationships among the three major placental branches Xenarthra, Afrotheria, and Boreotheria were intensively examined by two independent groups [15,16] that revealed markers for all possible variants of relationships, positive evidence supporting ancestral incomplete lineage sorting.

A third potential source of apparent conflicts in the presence/absence patterns of retroposed element insertions is ancestral hybridization, expressed by the exchange of genetic material between separated populations that are still able to reproduce with one another. After hybridization, a new lineage or mixed old lineages can evolve that carry different amounts of genetic material from both lineages. This might lead to asymmetric polytomy, as proposed for an overlapping retroposon distribution (e.g., two elements shared by guinea pig and squirrel vs. eight elements shared by mouse and guinea pig, but no elements shared between mouse and squirrel [17]).

Two other potential sources of conflicts, the exact deletion or parallel insertion of retroelements in related species, are both very rare (see also above). Lagemaat et al. [5] claimed to have found rare cases of exact deletions in young insertions with perfect recombining TSDs; however, the data are not distinguishable from those that might result from incomplete lineage sorting. Notable is, that any exact deletion or exact parallel insertion (producing the same TSDs) in individual genomes must spread over the population to finally be fixed in a lineage. So, random exact deletions or parallel insertions are very rare. For LINE1-mobilized retropositions, one can recognize a slight preference for a TT/AAAA target site motif [18] (the slash represents the cutting/insertion site) perhaps generating some slight hotspots for insertions. The distribution of such rare conflicting cases is only detectable in high-throughput computational or experimental screening for phylogenetic markers [19].

At nearly the same time that insertions of SINEs were proposed as phylogenetic markers [1], the probability of obtaining incorrect phylogenetic information due to segregation of ancestral polymorphism was intensively debated in the phylogenetic community [20] and ancestral polymorphism is now known to be common in lineage diversification [8]. The first consideration of polymorphic markers was based on the principle of Kimura’s neutral theory of molecular evolution [21]. However, in some of these early publications, the only source of phylogenetic conflicts considered was ancestral polymorphism due to incomplete lineage sorting [14,20,22]. Recently, polymorphism due to ancestral hybridization as source for conflicting phylogenetic resolutions was discussed [23,24] and illustrated at the sequence analysis level [25].

Notably, the probability of deriving incorrect phylogenetic signals from ancestral polymorphisms was first shown for rare and irreversible mutations [20], which can be adapted to the analysis of presence/absence of retroelements. Waddell et al. [26] created a criterion for supportive and/or conflicting SINE insertions to support or reject predefined phylogenetic topologies depending on a predefined prior hypothesis against polytomy due to incomplete lineage sorting. The use of this criterion became more popular with the rising popularity of the nearly conflict-free nature of presence/absence data and the increasing availability of genomic data. Nevertheless, from time to time apparently conflicting patterns were recovered and described (e.g., [27]). Unfortunately, the Waddell criterion [26] has many shortcomings that are not compatible with current requirements. For example, the restriction to only test trees limited to the support of five potential phylogenetically informative markers versus symmetric polytomies, or the requirement when testing experimental data that an equal amount of data must be testable for all three possible tree configurations of three species (e.g., for gorilla, chimpanzee, and human ideally an equal number of markers derived from all individual genomes should be screened) is often not available from *in silico* data. The current immense accumulation of genomic data facilitates novel multi-lineage perspectives to search for phylogenetically informative markers but also requires novel statistical models.

**Fig 1:**
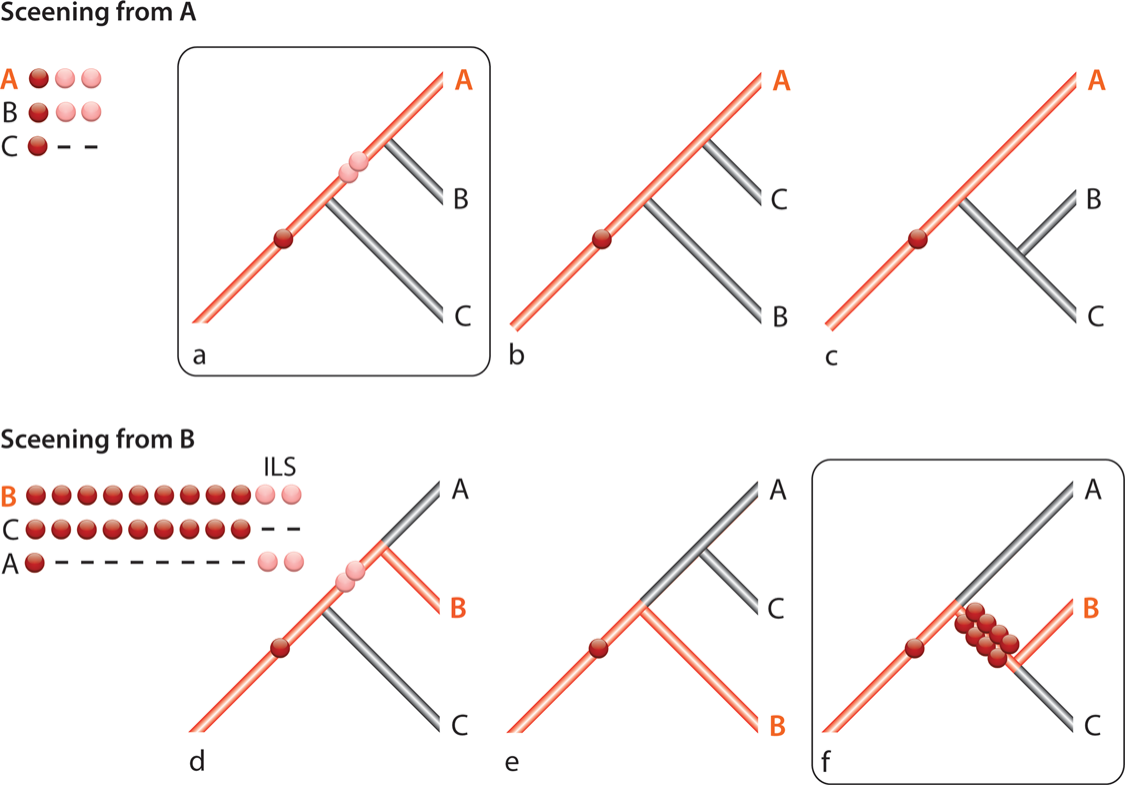
Possible discrepancy between one- and two-sided (species) screenings. Screening for phylogenetic markers based on all possible tree topologies for three species A, B, C when only one reference genome A (a-c) or B (d-f) is available. The red lineage indicates the branches where markers can be detected. Screening from A reveals three markers. The two light red markers are artifacts from ancient incomplete lineage sorting (ILS) and the dark red marker is a phylogenetically informative marker. Screening from B reveals 11 markers with 8 markers supporting B plus C and one marker supporting A plus (B plus C). The two light red markers in (d) are the same detected from species A in (a). The correct topology is shown in tree (f). This correct tree would not be detectible by screening only from the genome of species A.

We should also note that not every phylogenetic reconstruction based on retroposon insertion presence/absence patterns is derived in an unbiased way (e.g., those derived from one-directional searches when just one of three genomes is available for screening; see supportable branches in red for a species A restricted search in Fig 1A–1C). Previously, we were not able to test all possible tree topologies for those derived from one-directional searches. As an example, the first systematic screenings for phylogenetically informative retroposon markers in primates [28] used the only available genome information available at the time, human. Therefore, only branches leading to human could be tested and supported (similar to the lineage leading to A in Fig 1A–1C). Other relationships apart from the human lineage could only be examined by inspecting the few additional random insertions also present by chance in the sequenced loci. The ideal situation is to independently screen for markers from two leading lineages (see Fig 1; screening from species A and B) to find all diagnostic insertions and potential conflicting markers.

To overcome the various shortcomings of previous statistical applications and to successfully analyze data that is somewhat less than ideal, we present a new statistical approach that provides a clear test system to evaluate the significance of retroposon presence/absence data and to differentiate between clear bifurcations, incomplete lineage sorting (polytomy), and ancestral hybridization scenarios. This tool is especially important for the high-throughput applications of current and upcoming genome projects due to the unlimited number of testable markers obtained. The new differentiation for one- and multi-directional searches (data from 1 or 2 and more leading species) embedded in a user-friendly R-application enables us to apply the significance test to different screening strategies, and is also suitable for those cases when genomic species representation is not optimal.

This approach dissects phylogenetic trees into series of 3 lineages and evaluates their relationships individually with the KKSC statistics. A statistical evaluation of branch support can be obtained for most such phylogenetic questions, but in the case of ancient rapid radiations leading to so-called anomaly zones with random distributions of polymorphic markers often spread over many speciation events, such a simplification will not solve conflicts between multiple groups. To find phylogenetically diagnostic presence/absence insertion signals in such zones is currently impossible (see [29], [30]), because the noise (random signals) overlays any potential useful signal. The proposed three-lineage subdivision is not adequate for such complexities, but the underlying mathematical model is being used to derive a multi-lineage application to extract hidden phylogenetic signals from a mosaic of marker information. Luckily, although such anomaly zones do exist, most phylogenetic questions are simple and easy to solve with the current strategy.

## Methods

The unbiased collection of phylogenetically informative presence/absence markers by computational comparative screening (searching for presence/absence patterns in the available sequenced genomes) and/or experimental amplification of promising loci is one of the first steps in reconstructing the evolutionary relationships among species that for example can be easily supplemented by using the GPAC presence/absence finder applied on available multi-way alignments [31]. The next and essential stage is to determine the reliability of the derived presence/absence data. This includes both the careful alignment of individual loci to define the clear orthology of markers and the removal of all loci with partial deletions and non-exact parallel insertions. All verified orthologous markers are then submitted to statistical analysis to derive the support values for the branches of the given species tree. Mathematical models are necessary that consider different biological scenarios. Starting with assumptions based on a simplified situation of three existing lineages that might have arisen following three different scenarios, binary branching, polytomy, or ancestral hybridization, we provide the basic mathematical conditions to be considered (see S1 Appendix). We call the new statistics the KKSC insertion significance test.

### 1. Model assumptions

We consider three currently existing lineages A, B, C with a common ancestry, and inspect the presence/absence patterns for retroelements inserted at orthologous genomic loci in these lineages. The following events were selected to define phylogenetically informative markers:

ω_1_—an orthologous retroelement is present in a genomic locus of A and B but absent in C;
ω_2_—an orthologous retroelement is present in a genomic locus of A and C but absent in B;
ω_3_—an orthologous retroelement is present in a genomic locus of B and C but absent in A.

We consider the random variable *η*_*j*_ as the number of events ω_*j*_ (i.e., this variable reflects the number of presence/absence markers supporting the relatedness of two appointed lineages). If the total number of all markers consolidating any two lineages (*n*):

$$n=\eta_{1}+\eta_{2}+\eta_{3}$$ (1)

is fixed, then, in compliance with the proposed model (see S1 Appendix, S1.8), the random variables *η*_1,_ *η*_2,_ *η*_3_ are distributed according a polynomial distribution:

$$P\left(\eta_{1}=y_{1},\eta_{2}=y_{2},\eta_{3}=y_{3}\right)=\frac{n!}{y_{1}!y_{2}!y_{3}!}p_{1}^{y_{1}}p_{2}^{y_{2}}p_{3}^{y_{3}},\;(y_{1}+y_{2}+y_{3}=n),$$ (2)

where the parameters of polynomial distribution *p*_1_, *p*_2_, and *p*_3_ are determined depending on which of the three models are applied, for binary branching, polytomy, and ancestral hybridization, respectively.

### 2. Binary tree

**Fig 2:**
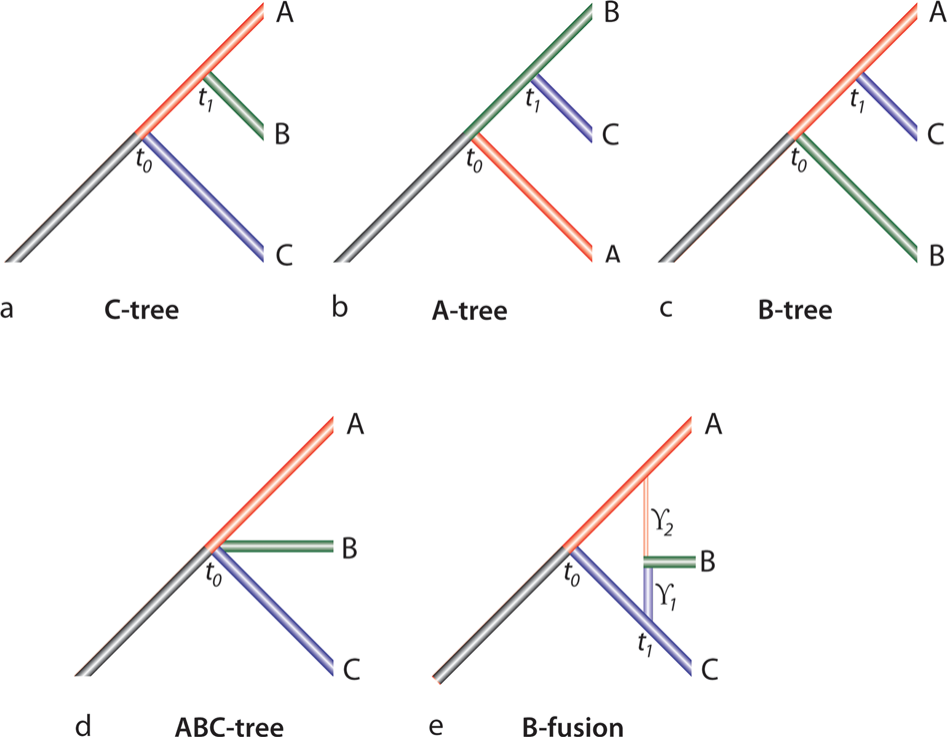
Schematic representation of various species trees. In all trees, lineage A is the red branch, lineage B is the green branch, and lineage C is the blue branch. (a) *C-tree*. First split: the ancestral population at time point *t*_*0*_ segregates into two branches, later one of them forms lineage C. At time point *t*_*1*_ the other branch diverges into the two lineages A and B. (b) *A-tree*. First split: the ancestral population at time point *t*_*0*_ segregates into two branches, later one of them forms lineage A. At time point *t*_*1*_ the other branch diverges into the two lineages B and C. (c) *B-tree*. First split: the ancestral population at time point *t*_*0*_ segregates into two branches, later one of them forms lineage B. At time point *t*_*1*_ the other branch diverges into the two lineages A and C. (d) *ABC-tree*. At the time point *t*_*0*_ the ancestral population segregates into three branches, later forming the three lineages A, B, and C (trifurcation). (e) Schematic representation of ancestral hybridization. *B-fusion*. First split: the ancestral population segregates at time point *t*_*0*_ into two branches. Subsequently, one of the branches (blue) splits after *t*_*1*_ generations, and the other branch (red) splits after *t*_*2*_ generations. The remaining parts of the blue and red lineages form lineages C and A, respectively. The derivates from the two joining populations form lineage B. The proportions of the parental populations forming lineage B are indicated by the coefficients γ_1_ and γ_2_, respectively.

Under the term *C-tree* we consider a scenario where at time *t*_0_ a common ancestral population separated into two isolated branches (that no longer interbreed). The first branch at time *T*_1_ (*t*_1_ = *t*_0_ + *T*_1_) subsequently separated into two lineages A and B. The second branch formed lineage C (Fig 2A).

In compliance with the proposed model (see also equations S1.38—S1.39 in S1 Appendix) we derive:

$$\left\{ \begin{array}{l} p_{1}=1-\frac{2}{3}\Psi \left(\tau_{1}\right)\\ p_{2}=p_{3}=\frac{1}{3}\Psi \left(\tau_{1}\right) \end{array}\right.,$$ (3)

where:

$$\tau_{1}=\frac{T_{1}}{2N_{1}}$$ (4)

is the drift time according to Waxman [41] (see equation S1.14 in S1 Appendix), and

$$\Psi (\tau )=\frac{e^{-\tau }}{1+\frac{n_{1}}{n_{0}}\left(\tau +e^{-\tau }-1\right)},$$ (5)

*N*_1_ is the average effective population size of the first branch before the split (at the period [*t*_0_, *t*_1_]), *n*_1_ is the average number of new insertions of retroelements per generation on this branch, and *n*_0_ is the average number of new insertions of retroelements per generation in an ancestral population.

It should be noted that formula (Eq 3) under condition *n*_1_ = *n*_0_ coincides with the formulations obtained by Wu [20] and corrected by Hudson [22] for a phylogenetic marker system, see also Liu [14].

Hence, the mathematical model for the *C-tree* corresponds to (Eq 2) under the assumption:

$$H_{1}=\left\{p_{2}=p_{3}=\frac{1-p_{1}}{2},p_{1}>\frac{1}{3}\right\}$$ (6)

Accordingly we can define the assumptions for the *B-tree* (Fig 2C):

$$H_{2}=\left\{p_{1}=p_{3}=\frac{1-p_{2}}{2},p_{2}>\frac{1}{3}\right\}$$ (7)

and the *A-tree* (Fig 2B):

$$H_{3}=\left\{p_{1}=p_{2}=\frac{1-p_{3}}{2},p_{3}>\frac{1}{3}\right\}.$$ (8)

Thus:

$$\begin{array}{l} P\left(\eta_{1}=y_{1},\eta_{2}=y_{2},\eta_{3}=y_{3}|H_{j}\right)=\frac{n!}{y_{1}!y_{2}!y_{3}!}p_{j}^{y_{j}}{\left(\frac{1-p_{j}}{2}\right)}^{n-y_{j}},\\ p_{j}>\frac{1}{3}\;,\;(y_{1}+y_{2}+y_{3}=n). \end{array}$$ (9)

An *ABC-tree* (polytomy) is the extreme form of an unresolved tree topology (Fig 2D):

$$H_{0}=\left\{p_{1}=p_{2}=p_{3}=\frac{1}{3}\right\},$$ (10)

that is:

$$P\left(\eta_{1}=y_{1},\eta_{2}=y_{2},\eta_{3}=y_{3}|H_{0}\right)=\frac{n!}{y_{1}!y_{2}!y_{3}!}\frac{1}{3^{n}}\;,\;(y_{1}+y_{2}+y_{3}=n).$$ (11)

If we assume that no other speciation scenario for A, B, and C is relevant, the parametric space for the model (Eq 2) reduces to:

$$\Omega =H_{0}\cup H_{1}\cup H_{2}\cup H_{3}.$$ (12)

Thus, to accept for example hypothesis *H*_1_, we must reject the opposite hypothesis:

$$H_{023}=H_{0}\cup H_{2}\cup H_{3}.$$ (13)

This leads to the fact that the data relevant for rejecting hypothesis *H*_023_ are at the same time sufficient for automatically accepting *H*_1_. An example result [27:13:0] representing relevant markers for the A, B, and C trees accordingly, will contradict the assumptions (Eq 7), (Eq 8), and (Eq 10) with a clear significance at the 5% level (in fact, even higher). This corresponds to Wu [20]. However, this result will also be inconsistent with (Eq 6) for the last two numbers [13:0] (for B and C trees). This indicates significant differences between *p*_2_ and *p*_3_ (that should be equal) that cannot be explained in either the present or previous models [20,22,26] or for coalescence models [14]. However, the skewed distribution of markers (e.g., 0 vs. 13) can be explained by ancestral hybridization [23,24]. To accommodate this, we added a simple model of hybridization that allows any combination of values of *p*_1_, *p*_2_, and *p*_3_, including the binary trees as a special case (see equations S1.40-S1.60 in S1 Appendix).

### 3. Ancestral hybridization

For ancestral hybridization (Fig 2E) we assume that at time *t* = *t*_0_ the common ancestral population separated into two isolated branches. Later, after *T*_*1*_ and *T*_*2*_ generations, subpopulations of each of the two branches separated from their parent branches (indicated by vertical lines on Fig 2E) and reproduce with one another, forming a new branch B (horizontal line, respectively; Fig 2E). The remaining two branches represent lineages A and C (Fig 2E). We will call this scenario *B-fusion*. In this simple scenario we ignore all events in the subpopulations before fusion, because elements inserted in genomes on these branches do not generate informative data.

The proportions of the two subpopulations in the newly joined population are denoted by γ_1_ and γ_2_ (γ_1_ + γ_2_ = 1). Then, according to the proposed mathematical model (equation S1.57 in S1 Appendix), if γ_1,2_ is not equal to 0 or 1 we have:

$$p_{1}>p_{2}\;\text{and}\;p_{3}>p_{2}.$$ (14)

When either γ_1_ or γ_2_ is equal to 0, we obtain an *A-tree* or *C-tree*, respectively. In the case of *C-fusion* (splits from A and B fuse), *p*_1_ exchanges places with *p*_2_, and in the case of *A-fusion* (splits from B and C fuse), *p*_3_ exchanges places with *p*_2_.

### 4. The statistical test

Consider the *C-tree* hypothesis:

$$H_{1}=\left\{p_{2}=p_{3}=\frac{1-p_{1}}{2},p_{1}>\frac{1}{3}\right\}.$$ (15)

In fact, this is equivalent to the two statements:

$$H_{1+}=\left\{p_{1}>\frac{1}{3}\right\}\;\text{and}\;H_{23}=\left\{p_{2}=p_{3}\right\}.$$ (16)

Therefore, *H*_1_ is accepted when both hypotheses (*H*_1+_ and *H*_23_) are supported and rejected when at least one of them is not accepted. In turn, the hypothesis *H*_1+_ is accepted when its opposite hypothesis ${\bar{H}}_{1+}=\left\{p_{1}\leq \frac{1}{3}\right\}$ is rejected. *η*_1_ is a sufficient statistic for the parameter *p*_1_, and distributes according to the binomial distribution:

$$P(\eta_{1}=k)=\left(\begin{array}{c} n\\ k \end{array}\right)p_{1}^{k}\cdot {\left(1-p\right)}^{n-k},$$ (17)

where $\left(\begin{array}{c} n\\ k \end{array}\right)=\frac{n!}{k!\left(n-k\right)!}$.

Thus, if we obtain *η*_1_ = *Y*_1_, the critical region for the hypothesis ${\bar{H}}_{1+}$ is the set of values greater or equal to *Y*_1_. Then:

$$P\left(\eta_{1}\geq Y_{1}\right)=\sum_{k=Y_{1}}^{n}\left(\begin{array}{c} n\\ k \end{array}\right)p_{1}^{k}\cdot {\left(1-p\right)}^{n-k}=I_{p_{1}}(Y_{1},n-Y_{1}+1),$$ (18)

where *I*_*p*_(*x*,*y*) is an incomplete beta function, which can also be expressed by the cumulative binomial distribution function:

$$P_{binom}\left(m,n,p\right)=\sum_{k=0}^{m}\left(\begin{array}{c} n\\ k \end{array}\right)p^{k}\cdot {\left(1-p\right)}^{n-k}=1-I_{p}(m+1,n-m).$$ (19)

Thus, the significance level is defined by the formula:

$$SL_{1}(Y)=\underset{p\leq \frac{1}{3}}{\underset{︸}{\max}}P(\eta_{1}\geq Y_{1})=I_{\frac{1}{3}}(Y_{1},n-Y_{1}+1).$$ (20)

We define the maximum probability of a *Type I Error* α as the probability to reject ${\bar{H}}_{1+}$in favor of *H*_1+_ when ${\bar{H}}_{1+}$ is true. Thus, if *SL*_1_(*Y*) ≤ *α*, then hypothesis ${\bar{H}}_{1+}$is rejected, and hypothesis *H*_1+_ is accepted.

Note, that when testing the hypothesis *H*_23_, the conditional distribution of the random variable *η*_2_ is binomially distributed with the parameter $p=\frac{p_{2}}{p_{2}+p_{3}}$:

$$P(\eta_{1}=k|\eta_{2}+\eta_{3}=m)=\left(\begin{array}{c} m\\ k \end{array}\right)p^{k}\cdot {\left(1-p\right)}^{m-k},$$ (21)

and hypothesis *H*_23_ is equivalent to the statement: $p=\frac{1}{2}$.

When using a two-sided test, the test statistics will be max{*η*_2_,*η*_3_}. In the case that the experimental data is validated (*η*_2_ = *Y*_2_, *η*_3_ = *Y*_3_), the critical region for the hypothesis *H*_23_ is the set of values {*y*_2_ + *y*_3_ = *Y*_2_ + *Y*_3_, max{*y*_2_,*y*_3_} ≥ max{*Y*_2_,*Y*_3_}}.

Accordingly, the level of significance is:

$$SL_{23}(Y)=|\begin{array}{l} 2I_{\frac{1}{2}}(\max{\left\{Y_{2},Y_{3}\right\}}_{1},\min\left\{Y_{2},Y_{3}\right\}+1),\;\text{if}\;Y_{2}\neq Y_{3},\\ 1,\;\text{if}\;Y_{2}=Y_{3}, \end{array}$$ (22)

**Fig 3:**
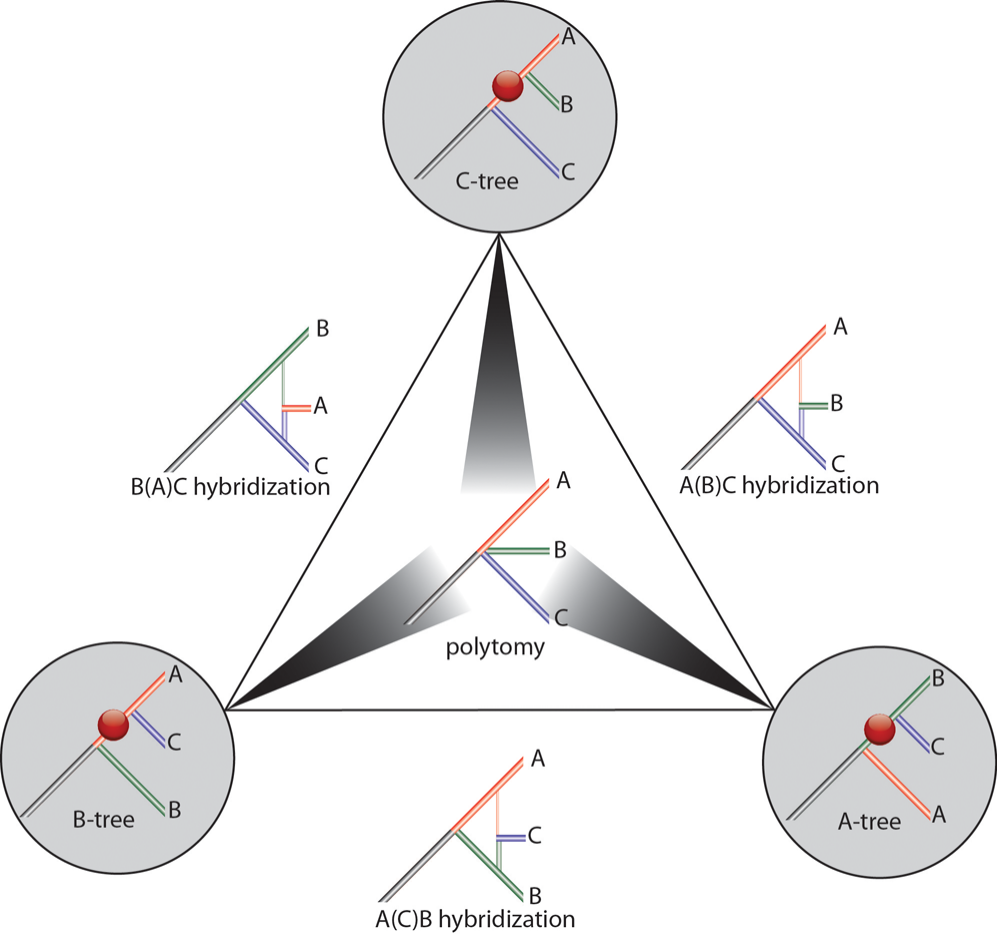
Schematic representation of all possible phylogenetic patterns. For the markers *n*_1_—(AB)C, *n*_*2*_—(AC)B, and *n*_*3*_—(BC)A, their sum *n* is fixed (*n = n*_*1*_*+n*_*2*_*+n*_*3*_). The triangle reflects all possible combinations of *n*_1_, *n*_*2*_, and *n*_*3*_, whereby the values at the corners are (*n*_*1*_:0:0), (0:*n*_*2*_:0), and (0:0:*n*_*3*_) (counterclockwise from the upper corner). The respective trees indicate supported tree configurations (*C-tree*, *A-tree*, and *B-tree*), red balls consolidate insertion support for the given branches. The grey scale arrowheads within the triangle indicate the statistically significant combinations of supporting tree configurations shown at the corners of the triangle; the darker the arrow the more significant support for the corresponding tree, the lighter the arrow the less support and the more the branching resembles a polytomy. The circular area at the center of the triangle denotes the *polytomy* zone (*ABC-tree*, where *n*_*1*_ = *n*_*2*_ = *n*_*3*_). The trees on the outside edges of the central triangle indicate *hybridization* zones (*B-fusion*, *C-fusion*, and *A-fusion*, denoted as A(B)C hybridization (where *n*_*1*_≥*n*_*2*_, and *n*_*2*_*>n*_*3*_), A(C)B hybridization (where *n*_*1*_≥*n*_*3*_, and *n*_*2*_<*n*_3_), and B(A)C hybridization (where *n*_*2*_≥*n*_*3*_, and *n*_*1*_<*n*_*2*_), respectively).

An illustration of all outcomes for the random distribution of markers and significance areas is presented in Fig 3.

In the case of a one-directional search for markers, data are only available to support two configurations of trees, while four configurations of trees and three hybridization scenarios are possible. This information is insufficient for our full model, but for the condition in which hybridization has already been ruled out, and we assume that only bifurcating trees or polytomy are possible, we derived a simple model for comparing two random binomial-distributed variables (see equations S1.61—S1.63 in S1 Appendix).

We consider the random binomial-distributed variables *η*_1_ and *η*_2_ and testing of hypothesis $H_{0}:p_{1}\leq \frac{1}{2}$ (*B-tree*, *A-tree*, *ABC-tree*) against an alternative hypothesis $H_{1}:p_{1}>\frac{1}{2}$ (*C-tree*). Then, when H_0_ is rejected because *Y*_1_ is significantly bigger than *Y*_2_, the *C-tree* can be accepted. In the opposite case, when *Y*_2_ > *Y*_1_, we can reject $H_{0}:p_{2}\leq \frac{1}{2}$ (*C-tree*, *A-tree*, *ABC-tree*) and accept the *B-tree*. If *Y*_1_ and *Y*_2_ are empirical values of *η*_1_ and *η*_2_, then, calculating similarly to (Eq 22), the level of significance will be:

$$SL(Y)=I_{\frac{1}{2}}(Y_{1},Y_{2}+1)$$ (23)

(Except in the situation where *Y*_1_ = *Y*_2_ and *H*_*0*_ is certainly accepted).

### 5. Approximations

The direct calculation of probabilities for large sets of phylogenetic markers requires some extensive calculations and extended knowledge of mathematical functions. Approximations can help to derive computational scripts including the statistical test. To find the boundaries of critical areas, we can use the normal approximation:

$$P(\eta_{1}\geq Y_{1})\approx 1-F_{0}\left(\frac{Y_{1}-\frac{1}{2}-np_{1}}{\sqrt{np_{1}(1-p_{1})}}\right),$$ (24)

where *F*_0_(*x*) is the standard normal distribution function. Denoting *z*_*α*_ as the root of the equation *F*_0_(*z*) = 1−*α*, from the condition *P*(*η*_1_ ≥ *Y*_1_) ≤ *α* and assuming $p_{1}=\frac{1}{3}$ we obtain:

$$Y_{1}\geq \frac{n}{3}+\frac{1}{2}+z_{\alpha }\frac{\sqrt{2n}}{3}.$$ (25)

Proceeding similarly, we define the second critical area for a given level of significance α as:

$$\left|Y_{2}-Y_{3}\right|\geq 1+z_{\frac{\alpha }{2}}\sqrt{Y_{2}+Y_{3}}.$$ (26)

In the case of a one-sided comparison (23), the critical area is defined by the formula:

$$Y_{1}\geq \frac{Y_{1}+Y_{2}+1+z_{\alpha }\sqrt{Y_{1}+Y_{2}}}{2}.$$ (27)

Values for *z*_*α*_ used in the approximated formulas (25), (26) and (27) are given in Table S1 in S2 Appendix for significance levels α<0.05, α<0.025, α<0.01, and α<0.005.

### 6. Implementation

The statistical model described here was implemented in a graphical web-application available at http://retrogenomics.uni-muenster.de:3838/KKSC_significance_test/. The application is generated with the Shiny package [32] in the R language [33]. No additional software needs to be installed to use it.

## Results

Based on our proposed mathematical model presented in the Methods (see also S1 Appendix), we can calculate the critical values for the numbers of markers shared by two lineages for various schemes of phylogenetic studies. A one-directional search (genome information of only one of three species is available; e.g., Fig 1A–1C for species A) provides a very limited amount of interpretable information. The calculation is based on formulas (Eqs 23 and 27) (see Table S2 in S3 Appendix). However, interpretations of presence/absence patterns derived from one-directional searches should be made with care. The lack of a difference between two values (numbers of markers) does not necessarily reject the third possible tree configuration, which cannot be tested from this one direction, and cannot exclude a polytomy between all three possible configurations or a significantly resolved third tree hypothesis (e.g., Fig 1F; the genome of species B is necessary). On the other hand, based on our model, differences between the two smallest values indicate ancestral hybridization events. Then significant statistical differences between two values obtained in the one-directional search do not distinguish between the possible bifurcated tree and hybridization (see equations S1.61—S1.63 in S1 Appendix).

In contrast to one-directional searches, unbiased screenings (multi-directional search) from two directions (e.g., Fig 1 using genomes of species A and B), returning three values for the numbers of shared markers, provide more information for interpretation (Tables S3-S4 in S3 Appendix), based on our statistical two-step criterion (Eqs 22–23). Using our web-interface and the implemented model (Eqs 20 and 22 and 23), we can easily derive P-values for the different phylogenetic scenarios (http://retrogenomics.uni-muenster.de:3838/KKSC_significance_test/).

An example of a conflicting distribution of markers was detected when we inspected the root of placental mammals [15]. We identified a presence/absence pattern of (9:8:5) similarly supporting all three possible tree hypotheses (Epitheria, Atlantogenata, and Exafroplacentalia). Using the web application to resolve this contradiction, the user first selects the “Analysis type”, either a “multi-directional” search (for cases in which more than one reference genome were screened, as in this example), or a “one-directional” search (for cases in which a screening was performed from only one reference species). It is also possible to specify the names of the species (e.g., A: Afrotheria; B: Xenarthra; C; Boreotheria), which are used for the results table. The user then provides the numbers of markers shared by the lineages that were analyzed. For the current example of a multi-directional analysis, the Afrotheria and Xenarthra shared 8 markers, Xenarthra and Boreotheria shared 5, and Afrotheria and Boreotheria 9 markers. The table at http://retrogenomics.uni-muenster.de:3838/KKSC_significance_test/ displays statistical information about the tests. The column “test type” displays the type of the test, and P-values are calculated for the different tests based on the values presented in the third column (e.g., p = 0.5811 for the hybridization test and p = 0.293 for bifurcation test). The fourth and fifth columns display the boundaries of critical areas for p<0.05 and p<0.01, respectively. The resulting figure of the KKSC significance test highlights the most probable evolutionary scenario. Significantly supported lineages are labeled by dark spheres; hybridization is indicated by divided spheres labeled with the hybridizing lineages; and the tree located in the center of the triangle indicates an unresolved tree topology.

We have also presented an applicable approximation for an unlimited number of markers (Eqs 25–27). As can be seen in Tables S3-S4 in S3 Appendix (columns 5% and 1% borders), this approximation effectively works from the minimum number of markers, and can be used as a brief estimation of significance of ongoing experimental results without using tables or the web-interface. For example, Nishihara et al. [16] examined the root of the placental tree and found 25 retroposon insertions supporting the Epitheria hypothesis, 22 supporting the Exafroplacentalia hypothesis, and 21 supporting the Atlantogenata hypothesis. Because the total number of markers is larger than 30, the pattern (25:22:21) cannot be directly evaluated using Tables S3-S4 in S3 Appendix. Therefore, to test the significance of the support for the various hypotheses the approximation formulas or the web-interface (http://retrogenomics.uni-muenster.de:3838/KKSC_significance_test/) should be used. To test the Epitheria hypothesis: calculate the sum of the relevant supporting markers (22+21 = 43) and the difference of the two smallest values (22–21). Setting the significance level at α<0.05, from Table S1 in S2 Appendix, we have a value of $z_{\frac{\alpha }{2}}=1.960$. Using equation (Eq 26) we can calculate the critical value for the difference of the two smallest values for their sum 43 and round this value up to the closest integer value: $1+1.960\cdot \sqrt{43}\approx 13.9=⊳14$. Thus, on the level of a significance of α<0.05, we cannot accept the hybridization hypothesis. To test the Epitheria hypothesis against polytomy we calculate the full sum (n = 25+22+21 = 68) and use equation (Eq 25). Setting the significance level at α<0.05, from Table S1 in S2 Appendix we have a value of *z*_*α*_ = 1.645. Calculating the critical value and rounding up, we have: $0.5+\frac{68+1.645\cdot \sqrt{2\cdot 68}}{3}\approx 29.6=⊳30$. Then, because 30<33, polytomy cannot be rejected and should represent the most realistic evolutionary scenario.

We also analyzed an interesting example of asymmetric conflicts in rodents. To determine the origin of the three major rodent lineages, best represented by mouse, guinea pig, and squirrel [16], we found 8 markers shared by mouse and guinea pig to the exclusion of squirrel, but also two markers shared by guinea pig and squirrel to the exclusion of mouse, and no insertions shared by mouse and squirrel. Because the Waddell criterion is limited to only 5 markers [26], it was not possible to use it to statistically evaluate this pattern. With our new statistical models we can test this case for significance of a resolved tree topology or hybridization. In the pattern (8:2:0), the two smallest values (2:0) do not fulfill the minimum number of markers for supporting a clear hybridization scenario (see Table S3 in S3 Appendix), so the critical values cannot be calculated and we cannot yet accept hybridization (p>0.05) as a viable hypothesis. According to our web-interface, a resolved tree topology of *(mouse*, *guinea pig)*, *squirrel* is supported at a significance level of p=0.0034. However, under our criteria, hybridization can only be significantly supported when there are 12 or more markers. This example shows that an appropriate statistical model plus a sufficient number of markers are necessary to correctly interpret hybridization signals.

Based on our mathematical model a calculation of the confidence intervals of drift time (*τ*) for a common ancestor of the two youngest lineages is possible (see S4 Appendix for details, examples, and simulation results).

## Discussion

The first phylogenetic applications of retroposon presence/absence patterns were conducted with a few hand-selected cases [34]. The clear polarity of retroposon markers, with presence as the derived condition and absence as the ancestral state, encountered little if any conflicting situations and designated retroposons as perfect, homoplasy-free markers [6]. As more and more genome data became available, seemingly conflicting patterns of markers were also obtained, requiring that we pay more careful attention to these conflicts in applying statistically meaningful tests. In addition to the conflicting retroposon presence/absence pattern at the root of placental mammals [15,16], there is also a series of conflicting retroposon presence/absence patterns in neoavian birds [29,35]. These patterns are probably due to the effects of incomplete lineage sorting because all possible phylogenetic topologies are represented more or less equally. Contradicting phylogenetic signals from retroposon presence/absence data were also detected in cichlid fishes [36] and turtles [37].

Given that we know that such conflicts reflect real evolutionary paths and not problematic data, these same conflicting patterns can provide valuable information about the first steps of new lineages after speciation. Distinguishing between equal and unequal polytomies provides unique information about potential ancient hybridization events. Retroposon insertions are very stable over time and point mutations have not critically reduced the recognizability of these signals over hundreds of millions of years. The cases of noise, introduced by parallel insertion [4] and precise deletion of retroelements [5], does not significantly influence the retroposon data, because of their rare appearance. Nevertheless, from time to time we receive an indication that parallel insertion or exact deletion cannot be completely ruled out, even if it is just a minor part of the collected data. For example, of more than 300 retroposon markers analyzed in the order Carnivora, three were highly inconsistent [4]. Although their insertion sites appeared highly orthologous, their locations in completely different parts of the phylogenetic tree clearly ruled out insertions in a common ancestor or incomplete lineage sorting in a local anomaly zone of the tree. Instead, they could be seen as real examples of parallel insertions of identical elements with identical target side duplications in distant parts of a phylogenetic tree. Likewise, the few loci containing retroposed elements under strong selective pressure do not influence presence/absence patterns, because selection does not selectively remove or insert complete copies in one or more lineages. Lineage-specific conserved versus non-conserved orthologous retroposon loci are only considered if a clear presence/absence state is recognizable in all investigated lineages. Thus, compared to other types of molecular markers, the very stable and recognizable nature of clear orthologous retroposon insertions preserves and provides important information about different scenarios of speciation events.

Initially, only SINE elements more close to the terminal mammalian branches were used as phylogenetic clade markers because they are more specific for a restricted group of species and rarely traverse the order levels in mammals [27]. Thus, retroposon presence/absence data were initially restricted to primates, rodents, lagomorphs, afrotherians, xenarthrans etc., and the interrelationships among these groups were not analyzed using SINE elements. This limitation was overcome by screening for Long INterspersed Elements (LINEs) and Long Terminal Repeats (LTRs) and using them similar to SINEs as phylogenetically informative markers [16,38]. With this expansion, it was possible to analyze deep mammalian branches. At that time, however, despite the newly available mouse genome, the human genome was still taken as the leading source of initial screening for potential informative markers. The current large number of available genomes provides numerous possibilities to further extend retroposon searches and provides excellent sources for investigating the tree of life.

Ongoing full genome screenings for retroposon presence/absence patterns can provide hundreds or even thousands of retroposon markers [3,39]. However, a subsequent clear individual confirmation of orthology by inspecting the element type and orientation, determining the exact identical insertion sites and target site duplications, and, if applicable, considering diagnostic truncations points, is essential to obtaining a noise-free dataset for further reliable investigations. One such example is presented in Doronina et al. [3], where the phylogenetic relationships of the three carnivore superfamilies (Ursoidea, Musteloidea, and Pinnipedia) were examined. Analysis based on a combined SINE and LINE dataset provided the pattern (192:74:60), where 192 markers reflected the consolidation of Pinnipedia and Musteloidea, 74 markers indicated a common ancestral branch for Ursoidea and Musteloidea, and 60 markers provided support for a Pinnipedia/Ursoidea clade. The resolved tree topology of (Pinnipedia, Musteloidea) Ursoidea was supported at a significance level of p<3.3×10^−21^ using the KKSC statistics (the small asymmetry of (74:60) did not indicate hybridization (p>0.2). This result confirms the most recent supertree analyses [40]. The detected zone of intense incomplete lineage sorting fits well with the proposed extensive radiation at the beginning of arctoid evolution [41].

In principle, and in addition to the branch support statistics, it is possible to calculate/simulate specific parameters of ancestral populations such as the effective populations size, but the random nature of marker fixation renders such values not as trustworthy as sequence-based calculations. Therefore, we only present some possible calculations in the S4 Appendix.

For small numbers of markers, the KKSC presence/absence statistics corresponds to the values of the previously established Waddell test [25] but returns less significant values in apparent marker conflict situations such as 3:1:0 (p = 0.111 vs. p = 0.0617, respectively) (see Table S9 in S5 Appendix). This is due to the consideration of more complex evolutionary scenarios, such as ILS and ancient hybridization in KKSC. Unfortunately, the Waddell test is only applicable for up to 5 markers. Compared to the PAUP*4.0b10 presence/absence data analysis [42] (irrev.up option of character transformation) as for example applied in Doronina et al. [2], the new statistics provides more reliable estimates of branch supports, especially for small numbers of markers. For example, in PAUP a single diagnostic insertion leads to a bootstrap value of 100, but more realistically is not significant in KKSC (possible *Type I Error* of the PAUP estimation). For small numbers of supporting markers, a Bayesian inference (MrBayes, Standard Discrete Model: binary; ctype irreversible; [43]), applied for example in Doronina et al. [2] lacks resolution (e.g., 2:0:0, polytomy in MrBayes). A chi-square test leads to results similar to those of KKSC. Applying the Yates’s correction for continuity (advised for small numbers) [44] to small sets of markers (1–3) leads to non-significant results. Finally, the KKSC test is the only test that not only rejects polytomy (trifuraction) but also detects hybridization signals and significantly extends the previously standard application presented by Waddell et al. [25].

Based on the principles of population genetics and the neutral theory of evolution, our statistical models create complete sets of criteria for testing all possible evolutionary scenarios for retroposon presence/absence data that are not randomly distributed during rapid radiations. One of the novelties of our model is the inclusion of a simple scenario for ancestral hybridization that is necessary for explaining asymmetric patterns of retroposon presence/absence insertions. Furthermore, our statistical criteria can be applied to any irreversible, largely neutrally evolving set of molecular markers (e.g., retroposon or indel presence/absence data) without any upper limitations on the size of the dataset. As discussed above, our new model is partially compatible with the criteria of Waddell et al. [26], but at the same time markedly enlarges the applicability for comprehensive datasets as they are generated today from genome-level analyses.

There are some natural limits in the acquisition of sufficient data and interpretation of the statistical significance using our model, mainly concerning low quality data, for example from one-directional searches (see Fig 1). For a one-directional search, we can only obtain resolution for two possible evolutionary scenarios. For the third possible tree, no data are available and consequently no safe statistical statement can be made. Furthermore, for such a limited screening, the hybridization probability cannot be calculated. A second limitation is that an evaluation of the level of hybridization is not yet available. However, one can imagine a hypothetical situation in which the relevant markers are distributed as (101:11:0), in which hybridization is supported with high significance (p<0.001), but support for the first tree topology is strong enough (101 marker) to favor this topology. One solution of this problem may be to define a *tree with hybridization* as a specific case and restrict *hybridization* cases to situations where we cannot define a clear topology, when the two highest values (*Y*_1_ and *Y*_2_) have no statistical difference (note: comparisons of the two highest values can be derived from our new web-interface (http://retrogenomics.uni-muenster.de:3838/KKSC_significance_test/) or can be performed with Eq 23 or the approximation formula 27). We intend to derive a more sensitive detection model for hybridization as soon as more retroposon presence/absence data are available for proven hybridization events, for example from plant phylogeny.

We have repeatedly stressed the need for extremely careful validation of the orthology of insertion markers and for only using those that fulfill very strict criteria. Is it possible that such strong filtering biases the dataset? Ascertainment biases can arise when filtered markers are not obtained from a random sample of the polymorphisms in the population of interest [45]. Even though our selections are very strict, they are still random. However, it should be mentioned that under special conditions an extreme reduction in the number of informative markers can occur from a large pool of potentially informative markers. For example, to validate the position of platypus in the tree of mammals by retroposon data [46], we screened ~90 thousand markers, but only three of them fulfilled all the criteria of orthology in such a deep mammalian branch. In such cases, we try to add screenings for additional types of elements active at the same time (SINEs, LINEs, LTRs etc.) to gain more information. Although the three markers were randomly selected and distributed over the full genomic expansion, it remains a theoretical possibility that they belong to a special subset of phylogenetically inconsistent loci, (e.g., a special subsets of markers that were incompletely sorted). That is why we advise, in addition to using as many sources of information as possible, it is best to screen genome-wide so as to obtain the largest number of markers possible. We recommend using optimized search criteria involving at least two different lineages in a multi-sided screening, and require a much higher burden of significance for markers resulting from a single-sided search with the warning that specific tree topologies cannot be resolved from such restricted searches.

Another current limitation is the restriction of our statistical test to combinations of three lineages, which is sufficient for most specific phylogenetic questions. Recently, however, large genome sequence analyses yielded multilevel conflicts in phylogenetic signals including many more than just three lineages with inconsistent markers [29,30,35]. We are currently in the process of developing a new statistic for specifically resolving such complex relationships resulting from extreme population expansions after bottlenecks and successive speciation periods that are much shorter than the time necessary for marker fixation (see for example the neoavian radiation 66 million years before [33,34]).

The minimum number of markers required for significant support of a selected tree hypothesis is three conflict-free markers detected via data derived from representatives of at least two or all three lineages [3:0:0], in agreement with Waddell et al. [26]. If only one representative of the three investigated lineages is available, five markers are required for significant support [5:0:0]. The statistical test that also considers conflicting patterns of markers can be taken from Table S2 in S3 Appendix (up to 30 markers can be tested) or from Table S3 together with Table S4 in S3 Appendix (up to 30 markers can be tested). In both cases significance values can be derived directly from formulas (Eqs 22 and 23) and our web-interface (http://retrogenomics.uni-muenster.de:3838/KKSC_significance_test/).

We have provided a comprehensive statistical framework for testing the significance of support for phylogenetic hypotheses derived from genome-level data and for evaluating possible retroposon presence/absence patterns for different evolutionary scenarios, including polytomy, incomplete lineage sorting, and ancestral hybridization. Finally, a reliable, adaptable calculation for the significance of support for phylogenetic trees derived from genome-wide retroposon presence/absence data is now available.

## Supporting Information

S1 Appendix Detailed description of the mathematical model. Contains sections: Model Assumptions; Binary tree; Ancestral hybridization; One-directional search; Supplementary References.
(PDF)

S2 Appendix Values of *Z*_*α*_ for different levels of significance *α*. Supplementary Table S1.
(PDF)

S3 Appendix Table of critical values for one-directional and unbiased searches. Supplementary Table S2. Example. Supplementary Tables S3-S4. Example.
(PDF)

S4 Appendix Confidence intervals for drift time*τ*. Contains sections: Estimation of the confidence intervals for the drift time; evaluation of confidence interval for the drift time; simulation of confidence intervals for the drift time τ in a diploid population; examples of calculation of Ne confidence intervals for ancestral lineages. Supplementary Figure S1 Fig; Supplemantary Tables S5-S8.
(PDF)

S5 Appendix Comparative evaluation of branch points. Supplementary Table S9.
(PDF)

## References

[1] Ryan SC, Dugaiczyk A (1989) Newly arisen DNA repeats in primate phylogeny. Proc Natl Acad Sci U S A 86: 9360–9364. 2480599 10.1073/pnas.86.23.9360 PMC298495

[2] Shimamura M, Yasue H, Ohshima K, Abe H, Kato H, et al (1997) Molecular evidence from retroposons that whales form a clade within even-toed ungulates. Nature 388: 666–670. 9262399 10.1038/41759

[3] Doronina L, Churakov G, Shi J, Brosius J, Baertsch R, et al (2015) Exploring Massive Incomplete Lineage Sorting in Arctoids (Laurasiatheria, Carnivora). Mol Biol Evol 32: 3194–3204. 10.1093/molbev/msv188 26337548

[4] Ray DA, Xing J, Salem AH, Batzer MA (2006) SINEs of a nearly perfect character. Syst Biol 55: 928–935. 17345674 10.1080/10635150600865419

[5] van de Lagemaat LN, Gagnier L, Medstrand P, Mager DL (2005) Genomic deletions and precise removal of transposable elements mediated by short identical DNA segments in primates. Genome Res 15: 1243–1249. 16140992 10.1101/gr.3910705 PMC1199538

[6] Shedlock AM, Okada N (2000) SINE insertions: powerful tools for molecular systematics. Bioessays 22: 148–160. 10655034 10.1002/(SICI)1521-1878(200002)22:2<148::AID-BIES6>3.0.CO;2-Z

[7] Peng Z, Elango N, Wildman DE, Yi SV (2009) Primate phylogenomics: developing numerous nuclear non-coding, non-repetitive markers for ecological and phylogenetic applications and analysis of evolutionary rate variation. BMC Genomics 10: 247 10.1186/1471-2164-10-247 19470178 PMC2693144

[8] Edwards SV (2009) Natural selection and phylogenetic analysis. Proc Natl Acad Sci U S A 106: 8799–8800. 10.1073/pnas.0904103106 19470454 PMC2690041

[9] Crow JF, Kimura M (1972) The effective number of a population with overlapping generations: a correction and further discussion. Am J Hum Genet 24: 1–10. 5012689 PMC1762154

[10] Kapitonov VV, Jurka J (2006) Self-synthesizing DNA transposons in eukaryotes. Proc Natl Acad Sci U S A 103: 4540–4545. 16537396 10.1073/pnas.0600833103 PMC1450207

[11] Hartig G, Churakov G, Warren WC, Brosius J, Makalowski W, et al (2013) Retrophylogenomics place tarsiers on the evolutionary branch of anthropoids. Sci Rep 3: 1756 10.1038/srep01756 23629008 PMC3639448

[12] Mailund T, Munch K, Schierup MH (2014) Lineage sorting in apes. Annu Rev Genet 48: 519–535. 10.1146/annurev-genet-120213-092532 25251849

[13] Schmitz J, Zischler H (2004) Molecular cladistic markers and the infraordinal phylogenetic relationships of primates In: Kay RF, Ross C, editors. Anthropoid Origins: New Visions. NY: Kluwer Academic Press pp. 57–69.

[14] Liu L, Yu L, Edwards SV (2010) A maximum pseudo-likelihood approach for estimating species trees under the coalescent model. BMC Evol Biol 10: 302 10.1186/1471-2148-10-302 20937096 PMC2976751

[15] Churakov G, Kriegs JO, Baertsch R, Zemann A, Brosius J, et al (2009) Mosaic retroposon insertion patterns in placental mammals. Genome Res 19: 868–875. 10.1101/gr.090647.108 19261842 PMC2675975

[16] Nishihara H, Maruyama S, Okada N (2009) Retroposon analysis and recent geological data suggest near-simultaneous divergence of the three superorders of mammals. Proc Natl Acad Sci U S A 106: 5235–5240. 10.1073/pnas.0809297106 19286970 PMC2655268

[17] Churakov G, Sadasivuni MK, Rosenbloom KR, Huchon D, Brosius J, et al (2010) Rodent evolution: back to the root. Mol Biol Evol 27: 1315–1326. 10.1093/molbev/msq019 20100942

[18] Jurka J (1997) Sequence patterns indicate an enzymatic involvement in integration of mammalian retroposons. Proc Natl Acad Sci U S A 94: 1872–1877. 9050872 10.1073/pnas.94.5.1872 PMC20010

[19] Nilsson MA, Klassert D, Bertelsen MF, Hallstrom BM, Janke A (2012) Activity of ancient RTE retroposons during the evolution of cows, spiral-horned antelopes, and Nilgais (Bovinae). Mol Biol Evol 29: 2885–2888. 22688946 10.1093/molbev/mss158

[20] Wu CI (1991) Inferences of species phylogeny in relation to segregation of ancient polymorphisms. Genetics 127: 429–435. 2004713 10.1093/genetics/127.2.429 PMC1204370

[21] Kimura M (1955) Solution of a Process of Random Genetic Drift with a Continuous Model. Proc Natl Acad Sci U S A 41: 144–150. 16589632 10.1073/pnas.41.3.144 PMC528040

[22] Hudson RR (1992) Gene trees, species trees and the segregation of ancestral alleles. Genetics 131: 509–513. 1644284 10.1093/genetics/131.2.509 PMC1205022

[23] Kubatko LS (2009) Identifying hybridization events in the presence of coalescence via model selection. Syst Biol 58: 478–488. 10.1093/sysbio/syp055 20525602

[24] Meng C, Kubatko LS (2009) Detecting hybrid speciation in the presence of incomplete lineage sorting using gene tree incongruence: a model. Theor Popul Biol 75: 35–45. 10.1016/j.tpb.2008.10.004 19038278

[25] Roos C, Zinner D, Kubatko LS, Schwarz C, Yang M, et al (2011) Nuclear versus mitochondrial DNA: evidence for hybridization in colobine monkeys. BMC Evol Biol 11: 77 10.1186/1471-2148-11-77 21435245 PMC3068967

[26] Waddell PJ, Kishino H, Ota R (2001) A phylogenetic foundation for comparative mammalian genomics. Genome Inform 12: 141–154. 11791233

[27] Shedlock AM, Takahashi K, Okada N (2004) SINEs of speciation: tracking lineages with retroposons. Trends Ecol Evol 19: 545–553. 16701320 10.1016/j.tree.2004.08.002

[28] Schmitz J, Ohme M, Zischler H (2001) SINE insertions in cladistic analyses and the phylogenetic affiliations of Tarsius bancanus to other primates. Genetics 157: 777–784. 11156996 10.1093/genetics/157.2.777 PMC1461532

[29] Matzke A, Churakov G, Berkes P, Arms EM, Kelsey D, et al (2012) Retroposon insertion patterns of neoavian birds: strong evidence for an extensive incomplete lineage sorting era. Mol Biol Evol 29: 1497–1501. 10.1093/molbev/msr319 22319163

[30] Suh A, Smeds L, Ellegren H (2015) The Dynamics of Incomplete Lineage Sorting across the Ancient Adaptive Radiation of Neoavian Birds. PLoS Biol 13: e1002224 10.1371/journal.pbio.1002224 26284513 PMC4540587

[31] Noll A, Raabe CA, Churakov G, Brosius J, Schmitz J (2015) Ancient traces of tailless retropseudogenes in therian genomes. Genome Biol Evol 7: 889–900. 10.1093/gbe/evv040 25724209 PMC5322556

[32] Chang W, Cheng J, Allaire JJ, Xie Y, Mac-Pherson J (2015) shiny: Web Application Framework for R.

[33] R.Core.Team (2014) R: A Language and Environment for Statistical Computing.

[34] Nikaido M, Rooney AP, Okada N (1999) Phylogenetic relationships among cetartiodactyls based on insertions of short and long interpersed elements: hippopotamuses are the closest extant relatives of whales. Proc Natl Acad Sci U S A 96: 10261–10266. 10468596 10.1073/pnas.96.18.10261 PMC17876

[35] Suh A, Paus M, Kiefmann M, Churakov G, Franke FA, et al (2011) Mesozoic retroposons reveal parrots as the closest living relatives of passerine birds. Nat Commun 2: 443 10.1038/ncomms1448 21863010 PMC3265382

[36] Takahashi K, Terai Y, Nishida M, Okada N (2001) Phylogenetic relationships and ancient incomplete lineage sorting among cichlid fishes in Lake Tanganyika as revealed by analysis of the insertion of retroposons. Mol Biol Evol 18: 2057–2066. 11606702 10.1093/oxfordjournals.molbev.a003747

[37] Sasaki T, Yasukawa Y, Takahashi K, Miura S, Shedlock AM, et al (2006) Extensive morphological convergence and rapid radiation in the evolutionary history of the family Geoemydidae (old world pond turtles) revealed by SINE insertion analysis. Syst Biol 55: 912–927. 17345673 10.1080/10635150601058014

[38] Kriegs JO, Churakov G, Kiefmann M, Jordan U, Brosius J, et al (2006) Retroposed elements as archives for the evolutionary history of placental mammals. PLoS Biol 4: e91 16515367 10.1371/journal.pbio.0040091 PMC1395351

[39] Platt RN 2nd, Zhang Y, Witherspoon DJ, Xing J, Suh A, et al (2015) Targeted Capture of Phylogenetically Informative Ves SINE Insertions in Genus Myotis. Genome Biol Evol 7: 1664–1675. 10.1093/gbe/evv099 26014613 PMC4494050

[40] Nyakatura K, Bininda-Emonds OR (2012) Updating the evolutionary history of Carnivora (Mammalia): a new species-level supertree complete with divergence time estimates. BMC Biol 10: 12 10.1186/1741-7007-10-12 22369503 PMC3307490

[41] Eizirik E, Murphy WJ, Koepfli KP, Johnson WE, Dragoo JW, et al (2010) Pattern and timing of diversification of the mammalian order Carnivora inferred from multiple nuclear gene sequences. Mol Phylogenet Evol 56: 49–63. 10.1016/j.ympev.2010.01.033 20138220 PMC7034395

[42] Swofford DL (2002) PAUP*: phylogenetic analysis using parsimony (*and other methods). 4.0b10.

[43] Ronquist F, Teslenko M, van der Mark P, Ayres DL, Darling A, et al (2002) MrBayes 3.2: efficient Bayesian phylogenetic inference and model choice across a large model space. Syst Biol 61: 539–542. 10.1093/sysbio/sys029 PMC3329765 22357727

[44] Yates F (1934) Contingency Tables Involving Small Numbers and the χ2 Test Supplement to the Journal of the Royal Statistical Society 1: 217–235.

[45] Heslot N, Rutkoski J, Poland J, Jannink JL, Sorrells ME (2013) Impact of marker ascertainment bias on genomic selection accuracy and estimates of genetic diversity. PLoS One 8: e74612 10.1371/journal.pone.0074612 24040295 PMC3764096

[46] Warren WC, Hillier LW, Marshall Graves JA, Birney E, Ponting CP, et al (2008) Genome analysis of the platypus reveals unique signatures of evolution. Nature 453: 175–183. 10.1038/nature06936 18464734 PMC2803040

