# Supplementary material for: Incomplete Lineage Sorting and Hybridization Statistics for Large-Scale Retroposon Insertion Data
Source: PLoS Comput Biol. 2016 Mar 11;12(3):e1004812. doi: 10.1371/journal.pcbi.1004812 (PMC4788455; doi:10.1371/journal.pcbi.1004812)
Supplement: S1 Appendix — Contains sections: Model Assumptions; Binary tree; Ancestral hybridization; One-directional search; Supplementary References. (PDF) [file pcbi.1004812.s001.pdf]

Detailed description of the mathematical model

Model Assumptions

Consider neutral insertions of retroelements in generation $t = s$ (t = time measured in generations). We take into account three different events:

ω_1 - an orthologous retroelement is present in a genomic locus of A and B but absent in C;

ω_2 - an orthologous retroelement is present in a genomic locus of A and C but absent in B;

ω_3 - an orthologous retroelement is present in a genomic locus of B and C but absent in A.

We denote: $q_1(s), q_2(s), q_3(s)$ as the probabilities of these events,

$\mu_1(s), \mu_2(s), \mu_3(s)$ as the numbers of these events,

$\nu(s)$ as the number of all insertions of retroelements in generation s.

Then, when we assume that all insertions are independent events, we have a scheme of independent trials with four outcomes distributed multinomially:

$$\mathrm{P}\big(\mu_1(s) = m_1, \mu_2(s) = m_2, \mu_3(s) = m_3 \big| \nu(s) = n\big) = \begin{cases} \dfrac{n!}{m_1!m_2!m_3!(n-m)!} q_1^{m_1} q_2^{m_2} q_3^{m_3} q_4^{n-m}, & if \;\; n \geq m, \\ 0, & if \;\; n < m \end{cases} \quad \text{(S1.1)}$$

where

$$\begin{aligned} q_4 &= q_4(s) = 1 - q_1(s) - q_2(s) - q_3(s), \\ m &= m_1 + m_2 + m_3 \end{aligned} \quad . \quad \text{(S1.2)}$$

For a shorter form of the equations, the dependence of probabilities on s is omitted. Hence, according to the total probability formula:

$$\begin{aligned} \mathrm{P}\left(\mu_1(s) = m_1, \mu_2(s) = m_2, \mu_3(s) = m_3\right) &= \sum_{n \geq m} \frac{n!}{m_1!m_2!m_3!(n-m)!} q_1^{m_1} q_2^{m_2} q_3^{m_3} q_4^{n-m} \mathrm{P}\left(\nu(s) = n\right) = \\ &= \frac{1}{m_1!m_2!m_3!} q_1^{m_1} q_2^{m_2} q_3^{m_3} \sum_{n \geq m} \frac{n!}{(n-m)!} q_4^{n-m} \mathrm{P}\left(\nu(s) = n\right) \end{aligned} \quad \text{(S1.3)}$$

If the probability of new insertions of retroelements in the genome of each particular member of the population $\alpha(s)$ is small, then we can assume that $\nu(s)$ is a Poisson distributed random variable with a mean $n_0 = n_0(s)$, proportional to the effective population size $N(s)$, that is:

$$\mathrm{P}\big(\nu(s) = n\big) = \frac{n_0^{\,n}}{n!} e^{-n_0} \quad \text{(S1.4)}$$

where $$n_0 = N(s)\alpha(s).$$

Thus

$$\sum_{n \geq m} \frac{n!}{(n-m)!} q_4^{n-m} \mathrm{P}\big(\nu(s) = n\big) = \sum_{k \geq 0} \frac{(q_4 n_0)^k}{k!} n_0^m e^{-n_0} = n_0^m e^{-(1-q_4)n_0}$$

therefore, denoting:

$$b_j = b_j(s) = n_0(s) q_j(s) \tag{S1.5}$$

we will get:

$$\mathrm{P}\left(\mu_1(s) = m_1, \mu_2(s) = m_2, \mu_3(s) = m_3\right) = \frac{1}{m_1! m_2! m_3!} b_1^{m_1} b_2^{m_2} b_3^{m_3} e^{-(b_1+b_2+b_3)}.$$

It follows that $\mu_1(s), \mu_2(s), \mu_3(s)$ represent independent, random, Poisson distributed variables with the parameters $b_1(s), b_2(s), b_3(s)$, respectively.

Now we consider all possible generations with potential retroposon insertions that later become phylogenetically informative. The set of corresponding values of time *s* we have denoted by *S*. Then the total numbers of retroposon insertions with properties ω_j :

$$\xi_j = \sum_{s \in S} \mu_j(s) \quad (j = 1, 2, 3)$$

are independent random, Poisson distributed variables with parameters:

$$a_j = \sum_{s \in S} b_j(s) = \sum_{s \in S} n_0(s) q_j(s). \tag{S1.6}$$

We consider the random variable η_j as the number of events ω_j observed in our experiment. If their total number:

$$\eta_1 + \eta_2 + \eta_3 = n \tag{S1.7}$$

is fixed, then, in compliance with the proposed model, the random variables $\eta_{1,}$ $\eta_{2,}$ η_3 are distributed according a polynomial distribution:

$$\mathrm{P}\left(\eta_1 = y_1, \eta_2 = y_2, \eta_3 = y_3\right) = \frac{n!}{y_1! y_2! y_3!} p_1^{y_1} p_2^{y_2} p_3^{y_3}, \ (y_1 + y_2 + y_3 = n) \tag{S1.8}$$

where

$$p_j = \frac{a_j}{a_1 + a_2 + a_3}. \tag{S1.9}$$

1. Binary tree

Under the term *C-tree* we consider a scenario where at time t_0 a common ancestral population separated into two isolated branches that no longer interbreed (Fig. 2a). The first branch, at some time T_1 ($t_1 = t_0 + T_1$), also separated into two lineages A and B. The second branch forms lineage C.

We take one certain marker locus (a locus in genomes containing an insertion of a retroelement). Denoting with $X(t)$ its frequency in the population at the time *t*, using the standard Wright-Fisher coalescent model ((Fisher 1922); (Wright 1931)), we can consider $X(t)$ as a Markov process with the transition function $u(s, p, t, x)$, reflecting the conditional probability density of $X(t)$

for condition $X(s) = p$. Under diffusion approximation, $u(s, p, t, x)$ obeys the forward Kolmogorov's equation:

$$\frac{\partial u}{\partial t} = \frac{1}{4N(t)} \frac{\partial^2}{\partial x^2} \left[x(1-x)u \right] , \tag{S1.10}$$

where the initial condition $u(s, p, s, x) = \delta(x - p)$ is a Dirac delta function, and $N(t) >> 1$ denotes the effective population size ((Kimura 1955a)).

The solution of this equation was first proposed by Kimura and afterwards in a global explanation by Tran et al. ((Tran, Hofrichter, and Jost 2013)) (for the case: $N(t)$ = const), and is represented in the form of a series including Gegenbauer polynomials. We do not use this solution because, for our purposes, it is sufficient to know some moments of distribution of $X(t)$.

We denote

$$m_k(s, p, t) = \int_0^1 x^k u(s, p, t, x) dx \tag{S1.11}$$

as the k-th (conditional) moment of distribution of $X(t)$ about 0.

Following Kimura (Kimura 1955b) we can write

$$m_1(s, p, t) = p \tag{S1.12}$$

and $m_k(s, p, t)$ is a solution of the next differential equation:

$$\frac{d}{dt} m_k(s, p, t) = -\frac{k(k-1)}{4N(t)} \left(m_k(s, p, t) - m_{k-1}(s, p, t) \right). \tag{S1.13}$$

Instead of t we introduce the new independent variable

$\tau = \tau(s,t) = \int_s^t \frac{dt}{2N(t)}$, as the "drift time", according to Waxman (Waxman 2011).(S1.14)

Then we can write equation (S1.13) in the form:

$$\frac{dm_k}{d\tau} = -\frac{k(k-1)}{2} \left(m_k - m_{k-1} \right) \tag{S1.15}$$

with an initial condition of: $m_k \big|_{\tau=0} = p^k$.

We also need the second and third moments. Solving the equation (S1.15) for k = 2 and k = 3, and taking into account (S1.12), we obtain:

$$m_2(s, p, t) = p - p(1-p)e^{-\tau(s,t)}. \tag{S1.16}$$

$$m_3(s, p, t) = p - \frac{3}{2} p(1-p)e^{-\tau(s,t)} + p(1-p)\left(\frac{1}{2} - p \right) e^{-3\tau(s,t)} \tag{S1.17}$$

(The same result may be obtained from the work of Kimura (Kimura 1955b) cited above, and also by using the solution of the diffusion equation proposed by Tran et al. ((Tran, Hofrichter, and Jost 2013)) if instead of *t*, we take the "drift time" τ).

As it follows from the Kimura notation, the conditional probability that the retroposon insertion, with a frequency at generation *s* equal to *p*, will be fixed in the population, tends to *p* with $t \to \infty$ (the probability of loss approaches 1 – *p*).

Consider some retroposon insertions into some loci in generation $s < t_0$. We introduce the random vector (X_0, X_1), where $X_0 = X(t_0), X_1 = X(t_1)$. With the fixed arbitrary values x_0, x_1 $(0 \le x_{0,1} \le 1)$, we can evaluate the conditional probabilities of $\omega_1, \omega_2, \omega_3$ accordingly:

$$\begin{aligned} &\mathrm{P}\left(\omega_1 \middle| X_0 = x_0, X_1 = x_1\right) = \left(1 - x_0\right) x_1^2, \\ &\mathrm{P}\left(\omega_2 \middle| X_0 = x_0, X_1 = x_1\right) = \mathrm{P}\left(\omega_3 \middle| X_0 = x_0, X_1 = x_1\right) = x_0 x_1 (1 - x_1) \end{aligned}. \qquad \text{(S1.18)}$$

Then, denoting $f(x_0, x_1)$ as the probability density of the random vector (X_0, X_1), the total probability formula will be:

$$\begin{aligned} &\mathrm{P}(\omega_1) = \int_0^1 \int_0^1 (1 - x_0) x_1^2 f(x_0, x_1) dx_0 dx_1, \\ &\mathrm{P}(\omega_2) = \mathrm{P}(\omega_3) = \int_0^1 \int_0^1 x_0 x_1 (1 - x_1) f(x_0, x_1) dx_0 dx_1 \end{aligned}, \qquad \text{(S1.19)}$$

and $f(x_0, x_1)$ transfers to:

$$f(x_0, x_1) = u(s, p, t_0, x_0) u(t_0, x_0, t_1, x_1), \qquad \text{(S1.20)}$$

where

$$p = \frac{1}{2N(s)}. \qquad \text{(S1.21)}$$

Thus

$$\begin{aligned} &\mathrm{P}(\omega_1) = \int_0^1 (1 - x_0) m_2(t_0, x_0, t_1) u(s, p, t_0, x_0) dx_0, \\ &\mathrm{P}(\omega_2) = \mathrm{P}(\omega_3) = \int_0^1 x_0 \left(m_1(t_0, x_0, t_1) - m_2(t_0, x_0, t_1)\right) u(s, p, t_0, x_0) dx_0 \end{aligned} \qquad \text{(S1.22)}$$

Hence:

$$\begin{aligned} &m_1(t_0, x_0, t_1) = x_0 \\ &m_2(t_0, x_0, t_1) = x_0 - x_0 (1 - x_0) e^{-\tau_1} \end{aligned}, \qquad \text{(S1.23)}$$

where

$$\tau_1 = \tau(t_0, t_1) = \int_{t_0}^{t_1} \frac{dt}{2N(t)}. \qquad \text{(S1.24)}$$

Then we obtain:

$$\begin{aligned} \mathrm{P}(\omega_1) &= \left(m_1(s,p,t_0) - m_2(s,p,t_0)\right)(1-e^{-\tau_1}) + \left(m_2(s,p,t_0) - m_3(s,p,t_0)\right)e^{-\tau_1}, \\ \mathrm{P}(\omega_2) &= \mathrm{P}(\omega_3) = \left(m_2(s,p,t_0) - m_3(s,p,t_0)\right)e^{-\tau_1}. \end{aligned} \qquad \text{(S1.25)}$$

Now, according to (S1.16-S1.17) with p defined by (S1.21) and neglecting the terms of order p^2 and higher (assuming that $N(t) >> 1$) we can write:

$$\begin{aligned} q_1(s) &= \mathrm{P}(\omega_1) = \left(1 - \frac{1}{2}e^{-\tau_1}\right) p e^{-\tau(s,t_0)} - \frac{1}{2}e^{-\tau_1} p e^{-3\tau(s,t_0)}, \\ q_2(s) &= q_3(t) = \mathrm{P}(\omega_2) = \mathrm{P}(\omega_3) = e^{-\tau_1}\frac{p}{2}\left(e^{-\tau(s,t_0)} - e^{-3\tau(s,t_0)}\right). \end{aligned} \qquad \text{(S1.26)}$$

Recall that $s \le t_0$. Suppose now that $t_0 < s \le t_1$ (a retroposon insertion occurs on branch 1). This marker will not appear in lineage C, hence $q_2(s) = q_3(s) = 0$. Let us evaluate $q_1(s) = \mathrm{P}(\omega_1)$. Noting that $\mathrm{P}(\omega_1 | X_1 = x_1) = x_1^2$, according to the total probability formula we get:

$$q_1(s) = \mathrm{P}(\omega_1) = \int_0^1 x_1^2 u(s,p,t_1,x_1)dx_1 = m_2(s,p,t_1). \qquad \text{(S1.27)}$$

Hence we finally can write:

$$\begin{aligned} q_1(s) &= p - pe^{-\tau(s,t_1)}, \\ q_2(s) &= q_3(s) = 0. \end{aligned} \qquad \text{(S1.28)}$$

(here $t_0 < s \le t_1$).

To find the parameters, a_1, a_2, a_3, according to (S1.9):

$$a_j = \sum_{s \in S} \alpha(s) N(s) q_j(s). \qquad \text{(S1.29)}$$

Assuming that the corresponding functions, as s increases by 1 (the transition to the next generation), are slowly changing, we replace the summation by an integration over the appropriate intervals. Then:

$$\begin{aligned} 2a_1 &= \left(1 - \frac{1}{2}e^{-\tau_1}\right)\int_{-\infty}^{t_0} \alpha(s) e^{-\tau(s,t_0)} ds - \frac{1}{2}e^{-\tau_1}\int_{-\infty}^{t_0} \alpha(s) e^{-3\tau(s,t_0)} ds + \int_{t_0}^{t_1} \alpha(s)\left(1 - e^{-\tau(s,t_1)}\right) ds, \\ 2a_2 &= 2a_3 = \frac{1}{2}e^{-\tau_1}\int_{-\infty}^{t_0} \alpha(s)\left(e^{-\tau(s,t_0)} - e^{-3\tau(s,t_0)}\right) ds. \end{aligned} \quad \text{, (S1.30)}$$

which, introducing the notation:

$$Z_1 = \int_{-\infty}^{t_0} \alpha(s) e^{-\tau(s,t_0)} ds,$$

$$Z_2 = \int_{-\infty}^{t_0} \alpha(s) e^{-3\tau(s,t_0)} ds, \qquad , \tag{S1.31}$$

$$Z_3 = \int_{t_0}^{t_1} \alpha(s)\left(1 - e^{-\tau(s,t_1)}\right) ds,$$

can be written:

$$a_1 = \left(1 - \frac{1}{2}e^{-\tau_1}\right) Z_1 - \frac{1}{2}e^{-\tau_1} Z_2 + Z_3,$$

$$a_2 = a_3 = \frac{1}{2}e^{-\tau_1}\left(Z_1 - Z_2\right), \tag{S1.32}$$

and now, similarly to Kimura (Kimura 1955b; Kimura 1955a), we assume for the all intervals $\alpha(t)$ a constant effective population size. Then, with $s \le t_0$:

$$\tau(s,t_0) = \frac{t_0 - s}{2N_0} \quad \text{and} \quad \left| \begin{array}{l} Z_1 = 2N_0\alpha_0 = n_0 \\ Z_2 = \frac{2}{3}N_0\alpha_0 = \frac{1}{3}n_0 \end{array} \right. , \; , \tag{S1.33}$$

where $n_0 = 2N_0\alpha_0$ is the average number of insertions per generation at the ancestral branch and N_0 is the effective population size.

Next, with $t_0 < s \le t_1$:

$$\begin{array}{l} \tau(s,t_1) = \frac{t_1 - s}{2N_1}, \\ \tau_1 = \tau(t_0,t_1) = \frac{T_1}{2N_1} \end{array} \quad \text{and} \quad Z_3 = \alpha_1\left(T_1 - 2N_{1e}\left(1 - e^{-\tau_1}\right)\right) = n_1\left(\tau_1 - 1 + e^{-\tau_1}\right), \tag{S1.34}$$

where $n_1 = 2N_1\alpha_1$ is the average number of insertions per generation at the branch 0-1 and N_1 the effective population size of this branch. Thus, introducing the function:

$$\Phi(\tau) = \tau - 1 + e^{-\tau}, \tag{S1.35}$$

according to (S1.32), we obtain:

$$a_1 = \left(1 - \frac{2}{3}e^{-\tau_1}\right) n_0 + n_1\Phi(\tau_1),$$

$$a_2 = a_3 = \frac{1}{3}e^{-\tau_1} n_0 . \tag{S1.36}$$

Now, following (S1.9):

$$p_1 = \frac{1 - \frac{2}{3} e^{-\tau_1} + \frac{n_1}{n_0}\Phi(\tau_1)}{1 + \frac{n_1}{n_0}\Phi(\tau_1)},$$

$$p_2 = p_3 = \frac{1}{3}\frac{e^{-\tau_1}}{1 + \frac{n_1}{n_0}\Phi(\tau_1)}, \tag{S1.37}$$

or, denoting

$$\Psi(\tau) = \frac{e^{-\tau}}{1 + \frac{n_0}{n_1}\left(\tau + e^{-\tau} - 1\right)}, \tag{S1.38}$$

we have

$$p_1 = 1 - \frac{2}{3}\Psi(\tau_1),$$

$$p_2 = p_3 = \frac{1}{3}\Psi(\tau_1)\,. \tag{S1.39}$$

Hence $p_1 > \frac{1}{3}$ and $p_2 = p_3 < \frac{1}{3}$ where $\Psi(\tau) < 1$.

2. Ancestral hybridization

Let us now consider a model that includes ancestral hybridization (Fig. 2e). As in the previous case we assume that at time t=t_0 the common ancestral population (branch 0) separated into two isolated branches. Later, after T_1 and T_2 generations, subpopulations of each of the two branches separated from their parent branches and reproduced with one another by fusion, forming a new branch B. The original two separating branches represent lineages A and C.

The proportions of the two subpopulations in the newly joined population are denoted by γ_1 and γ_2 ($\gamma_1 + \gamma_2 = 1$). Then:

$$\begin{aligned} &N_{13} = \gamma_1 N_3, N_{23} = \gamma_2 N_3, \\ &N_{13} + N_{23} = N_3 \end{aligned} . \qquad \text{(S1.40)}$$

Consider a retroposon insertion at a specific locus at $s < t_0$. We introduce the random vector $\left(X_0, X_1, X_2\right)$, where $X_0 = X(t_0), X_1 = X_{01}(t_1), X_2 = X_{02}(t_2)$. Fixing the arbitrary values x_0, x_1, x_2 $\left(0 \le x_{0,1,2} \le 1\right)$, we can write the conditional probabilities of the events $\omega_1, \omega_2, \omega_3$ respectively:

$$\begin{aligned} &\mathrm{P}\left(\omega_1 \middle| X_1 = x_1, X_2 = x_2\right) = x_1\left(\gamma_1 x_1 + \gamma_2 x_2\right)\left(1 - x_2\right), \\ &\mathrm{P}\left(\omega_2 \middle| X_1 = x_1, X_2 = x_2\right) = x_1\left(\gamma_1 (1 - x_1) + \gamma_2 (1 - x_2)\right) x_2 \, . \\ &\mathrm{P}\left(\omega_3 \middle| X_1 = x_1, X_2 = x_2\right) = (1 - x_1)\left(\gamma_1 x_1 + \gamma_2 x_2\right) x_2 \end{aligned} \qquad \text{(S1.41)}$$

Then, denoting $f\left(x_0, x_1, x_2\right)$ as the probability density of the random vector $\left(X_0, X_1, X_2\right)$, according to the total probability formula we get:

$$\begin{aligned} &\mathrm{P}\left(\omega_1\right) = \int_0^1 \int_0^1 \int_0^1 x_1\left(\gamma_1 x_1 + \gamma_2 x_2\right)\left(1 - x_2\right) f(x_0, x_1, x_2) dx_0 dx_1 dx_2, \\ &\mathrm{P}\left(\omega_2\right) = \int_0^1 \int_0^1 \int_0^1 x_1\left(\gamma_1 (1 - x_1) + \gamma_2 (1 - x_2)\right) x_2 f(x_0, x_1, x_2) dx_0 dx_1 dx_2, \\ &\mathrm{P}\left(\omega_3\right) = \int_0^1 \int_0^1 \int_0^1 (1 - x_1)\left(\gamma_1 x_1 + \gamma_2 x_2\right) x_2 f(x_0, x_1, x_2) dx_0 dx_1 dx_2. \end{aligned} \qquad \text{(S1.42)}$$

$f(x_0, x_1, x_2)$ transfers to:

$$f\left(x_0, x_1, x_2\right) = u_0(s, p, t_0, x_0) u_1(t_0, x_0, t_1, x_1) u_2(t_0, x_0, t_2, x_2), \qquad \text{(S1.43)}$$

where $u_0(s, p, t_0, x_0), u_1(t_0, x_0, t_1, x_1), u_2(t_0, x_0, t_2, x_2)$ are transitional functions for the respective branches.

Note that using the relations (S1.12) and (S1.16) we can write:

$$\begin{aligned}&\int_0^1 x_j u_j(t_0,x_0,t_j,x_j)dx_j = m_1^{(j)}(t_0,x_0,t_j) = x_0,\\ &\int_0^1 x_j^2 u_j(t_0,x_0,t_j,x_j)dx_j = m_2^{(j)}(t_0,x_0,t_j) = x_0 - x_0(1-x_0)e^{-\tau_j},\end{aligned}, \qquad \text{(S1.44)}$$

where

$$\tau_j = \int_{t_0}^{t_j} \frac{dt}{2N_j(t)}, \quad j\in\{1,2\}. \qquad \text{(S1.45)}$$

Hence:

$$\begin{aligned}
&\mathrm{P}(\omega_1) = \int_0^1 \left(\gamma_1\left(x_0 - x_0(1-x_0)e^{-\tau_1}\right)(1-x_0) + \gamma_2 x_0^2(1-x_0)e^{-\tau_2}\right)u(s,p,t_0,x_0)dx_0 =\\
&= \gamma_1\left((m_1-m_2)(1-e^{-\tau_1}) + (m_2-m_3)e^{-\tau_1}\right) + \gamma_2(m_2-m_3)e^{-\tau_2},\\
&\mathrm{P}(\omega_2) = \int_0^1 \left(\gamma_1 x_0^2(1-x_0)e^{-\tau_1} + \gamma_2 x_0^2(1-x_0)e^{-\tau_2}\right)u(s,p,t_0,x_0)dx_0 =\\
&= \left(\gamma_1 e^{-\tau_1} + \gamma_2 e^{-\tau_2}\right)(m_2-m_3),\\
&\mathrm{P}(\omega_3) = \int_0^1 \left(\gamma_1 x_0^2(1-x_0)e^{-\tau_1} + \gamma_2\left(x_0 - x_0(1-x_0)e^{-\tau_2}\right)(1-x_0)\right)u(s,p,t_0,x_0)dx_0 =\\
&= \gamma_1(m_2-m_3)e^{-\tau_1} + \gamma_2\left((m_1-m_2)(1-e^{-\tau_2}) + (m_2-m_3)e^{-\tau_2}\right),
\end{aligned} \qquad \text{(S1.46)}$$

wherein, using (S1.16)-(S1.17) and neglecting the terms of order p^2 and higher:

$$\begin{aligned}
&m_1 = m_1(t,p,t_0) = p\\
&m_2 = m_2(s,p,t_0) = p - pe^{-\tau(s,t_0)}.\\
&m_3 = m_3(s,p,t_0) = p - \frac{3}{2}pe^{-\tau(s,t_0)} + \frac{1}{2}pe^{-3\tau(s,t_0)}
\end{aligned} \qquad \text{(S1.47)}$$

Thus:

$$\begin{aligned}
&q_1(s) = \mathrm{P}(\omega_1) = \gamma_1\left(\left(1-\frac{1}{2}e^{-\tau_1}\right)pe^{-\tau(s,t_0)} - \frac{1}{2}e^{-\tau_1}pe^{-3\tau(s,t_0)}\right) + \gamma_2 e^{-\tau_2}\left(e^{-\tau(s,t_0)} - e^{-3\tau(s,t_0)}\right)\frac{p}{2},\\
&q_2(s) = \mathrm{P}(\omega_2) = \left(\gamma_1 e^{-\tau_1} + \gamma_2 e^{-\tau_2}\right)\left(e^{-\tau(s,t_0)} - e^{-3\tau(s,t_0)}\right)\frac{p}{2},\\
&q_3(s) = \mathrm{P}(\omega_3) = \gamma_1\left(e^{-\tau(s,t_0)} - e^{-3\tau(s,t_0)}\right)\frac{p}{2}e^{-\tau_1} + \gamma_2\left(\left(1-\frac{1}{2}e^{-\tau_2}\right)pe^{-\tau(s,t_0)} - \frac{1}{2}e^{-\tau_2}pe^{-3\tau(s,t_0)}\right),
\end{aligned} \qquad \text{(S1.48)}$$

(here $s \le t_0$).

Note that when $\gamma_1 = 1, \gamma_2 = 0$ (*C-tree* (see equation 6 in the Manuscript), Fig 2a) this result coincides with (S1.29), and for $\gamma_1 = 0, \gamma_2 = 1$ (*A-tree* (see equation 8 in the Manuscript), Fig 2b) we obtain similar formulas, where $q_1(s)$ is replaced by $q_3(s)$, and τ_1 is replaced by τ_2.

Suppose now that a retroposon insertion occurs on the branch 0-1 at $s \in (t_0, t_1)$. Then, it will not appear in the lineage C, and hence $q_2(s) = q_3(s) = 0$. We evaluate $q_1(s) = \mathrm{P}(\omega_1)$. Noticing that $\mathrm{P}(\omega_1 | X_1 = x_1) = \gamma_1 x_1^2$, the corresponding result obtained by multiplying the right side of equation (S1.28) by γ_1. Thus:

$$\begin{aligned} q_1(s) &= \gamma_1 \left(p - pe^{-\tau(s,t_1)}\right), \\ q_2(s) &= q_3(s) = 0 \end{aligned} \tag{S1.49}$$

Here $t_0 < s \le t_1$,

$$p = \frac{1}{2N_1(s)} \tag{S1.59}$$

and

$$\tau(s,t_1) = \int_s^{t_1} \frac{dt}{2N_1(t)}. \tag{S1.51}$$

Processing similarly with the retroposon inserted on branch 0-2, we obtain:

$$\begin{aligned} q_3(s) &= \gamma_2 \left(p - pe^{-\tau(s,t_2)}\right), \\ q_1(s) &= q_2(s) = 0 \end{aligned} \tag{S1.52}$$

Here $t_0 < s \le t_2$

$$p = \frac{1}{2N_2(s)} \tag{S1.53}$$

and

$$\tau(s,t_2) = \int_s^{t_1} \frac{dt}{2N_2(t)}. \tag{S1.54}$$

Next proceeding as in (S1.29 - S1.36), we can write:

$$\begin{aligned} a_1 &= \left(\left(1 - \frac{2}{3}e^{-\tau_1}\right)n_0 + n_1\Phi(\tau_1)\right)\gamma_1 + \frac{n_0}{3}e^{-\tau_2}\gamma_2 \\ a_2 &= \frac{n_0}{3}\left(e^{-\tau_1}\gamma_1 + e^{-\tau_2}\gamma_2\right) \\ a_3 &= \frac{n_0}{3}e^{-\tau_1}\gamma_1 + \left(\left(1 - \frac{2}{3}e^{-\tau_2}\right)n_0 + n_2\Phi(\tau_2)\right)\gamma_2 \end{aligned} \tag{S1.55}$$

Now, according to (S1.9):

$$p_1 = \frac{\left(1-\frac{2}{3}e^{-\tau_1}+\frac{n_1}{n_0}\Phi(\tau_1)\right)\gamma_1+\frac{1}{3}e^{-\tau_2}\gamma_2}{1+\frac{n_1}{n_0}\Phi\left(\tau_1\right)\gamma_1+\frac{n_2}{n_0}\Phi\left(\tau_2\right)\gamma_2},$$

$$p_2 = \frac{1}{3}\frac{e^{-\tau_1}\gamma_1+e^{-\tau_2}\gamma_2}{1+\frac{n_1}{n_0}\Phi\left(\tau_1\right)\gamma_1+\frac{n_2}{n_0}\Phi\left(\tau_2\right)\gamma_2}, \quad \text{(S1.56)}$$

$$p_3 = \frac{\frac{1}{3}e^{-\tau_1}\gamma_1+\left(1-\frac{2}{3}e^{-\tau_2}+\frac{n_2}{n_0}\Phi(\tau_2)\right)\gamma_2}{1+\frac{n_1}{n_0}\Phi\left(\tau_1\right)\gamma_1+\frac{n_2}{n_0}\Phi\left(\tau_2\right)\gamma_2}.$$

When either γ_1 or γ_2 are equal to 0, we obtain an *A-tree* ((see equation 8 in the Manuscript), Fig 2b) or a *C-tree* ((see equation 6 in the Manuscript), Fig 2a), respectively.

Note that:

$$\begin{aligned} a_1 - a_2 &= \left(\left(1-e^{-\tau_1}\right)n_0 + n_1\Phi(\tau_1)\right)\gamma_1 \\ a_3 - a_2 &= \left(\left(1-e^{-\tau_2}\right)n_0 + n_2\Phi(\tau_2)\right)\gamma_2 \end{aligned}. \quad \text{(S1.57)}$$

If $\gamma_{1,2}$ are not equal to 0, $a_1 > a_2$ and $a_3 > a_2$ (accordingly: $p_1 > p_2$ and $p_3 > p_2$). In the case of *C-fusion* (splits from A and B fuse), p_1 will exchange places with p_2, and in the case of *A-fusion* (splits from B and C fuse), p_3 will exchange places with p_2.

Consider the case:

$$\left(1-e^{-\tau_1}\right)n_0 + n_1\Phi(\tau_1) = \left(1-e^{-\tau_2}\right)n_0 + n_2\Phi(\tau_2) \quad \text{(S1.58)}$$

(this holds in particular if $n_1 = n_2 = n_0,\ \tau_1 = \tau_2$). Then:

$$p_1 = \frac{\left(1-\frac{2}{3}e^{-\tau}+\Phi\left(\tau\right)\right)\gamma_1+\frac{1}{3}e^{-\tau}\gamma_2}{1+\Phi\left(\tau\right)},$$

$$p_2 = \frac{1}{3}\frac{e^{-\tau}}{1+\Phi\left(\tau\right)} \quad \text{(S1.59)}$$

$$p_3 = \frac{\frac{1}{3}e^{-\tau}\gamma_1+\left(1-\frac{2}{3}e^{-\tau}+\Phi\left(\tau\right)\right)\gamma_2}{1+\Phi\left(\tau\right)}$$

These equations can also be written as:

$$p_1 = (1-2p_2)\gamma_1 + p_2\gamma_2,$$
$$p_2 = \frac{1}{3}\frac{e^{-\tau}}{1+\Phi(\tau)}, \tag{S1.60}$$
$$p_3 = p_2\gamma_1 + (1-2p_2)\gamma_2.$$

3. One-directional search

Now we consider the case when only two events, ω_1 and ω_2, can be observed. Thus there are only two random variables: η_1 and η_2. If their total number

$$\eta_1 + \eta_2 = n \tag{S1.61}$$

is fixed, then we have a binomial distribution:

$$\mathrm{P}(\eta_1 = y_1, \eta_2 = y_2) = \frac{n!}{y_1! y_2!} p_1^{y_1} p_2^{y_2}, \ (y_1 + y_2 = n), \tag{S1.62}$$

where

$$p_1 = \frac{a_1}{a_1 + a_2}, \ \ p_2 = \frac{a_2}{a_1 + a_2} \ \ (p_1 + p_2 = 1). \tag{S1.63}$$

In the case of a *C-tree,* according to (S1.36, 6) $a_1 > a_2$, hence $p_1 > \frac{1}{2}$.

Similarly for a *B-tree* (see equation 7 in the Manuscript, Fig. 2b) $a_1 < a_2$, therefore $p_1 < \frac{1}{2}$.

The case of the *A-tree* (see equation 8 in Manuscript) leads to $p_1 = \frac{1}{2}$, but it is necessary to note that the same situation occurs when we have an *ABC-tree* (see equation10 in the Manuscript, Fig. 2d) (polytomy). In the case of *B-fusion,* in accordance with the remark following (S1.57), we also have $p_1 > \frac{1}{2}$ and in the case of *C-fusion* $p_1 < \frac{1}{2}$. However, for *A-fusion,* the relationship between p_1 and p_2 may be arbitrary.

Supplementary References

Fisher, R. A. 1922. On the dominance ratio. P Roy Soc Edinb **42**:321-241.
Kimura, M. 1955a. Stochastic processes and distribution of gene frequencies under natural selection. Cold Spring Harb Symp Quant Biol **20**:33-53.
Kimura, M. 1955b. Solution of a Process of Random Genetic Drift with a Continuous Model. Proc Natl Acad Sci U S A **41**:144-150.
Tran, T. D., J. Hofrichter, and J. Jost. 2013. An introduction to the mathematical structure of the Wright-Fisher model of population genetics. Theory Biosci **132**:73-82.
Waxman, D. 2011. A unified treatment of the probability of fixation when population size and the strength of selection change over time. Genetics **188**:907-913.
Wright, S. 1931. Evolution in Mendelian Populations. Genetics **16**:97-159.
