# Supplementary material for: Incomplete Lineage Sorting and Hybridization Statistics for Large-Scale Retroposon Insertion Data
Source: PLoS Comput Biol. 2016 Mar 11;12(3):e1004812. doi: 10.1371/journal.pcbi.1004812 (PMC4788455; doi:10.1371/journal.pcbi.1004812)
Supplement: S2 Appendix — Supplementary Table S1. (PDF) [file pcbi.1004812.s002.pdf]

Values of Z_α for different levels of significance α.

Table S1. Values of Z_α for different levels of significance α.

α	0.005	0.01	0.025	0.05
Z_α	2.576	2.326	1.960	1.645
