# Supplementary material for: Incomplete Lineage Sorting and Hybridization Statistics for Large-Scale Retroposon Insertion Data
Source: PLoS Comput Biol. 2016 Mar 11;12(3):e1004812. doi: 10.1371/journal.pcbi.1004812 (PMC4788455; doi:10.1371/journal.pcbi.1004812)
Supplement: S3 Appendix — Supplementary Table S2. Example. Supplementary Tables S3-S4. Example. (PDF) [file pcbi.1004812.s003.pdf]

Tables of critical values for one-directional and unbiased searches

Testing: one-directional search

To determine if the number of phylogenetically informative markers on a given branch is sufficient to provide significant support for one hypothesis or another, the evaluation of the results of a biased, one-directional search using this table (Table S2) includes the following steps: (1) calculating the sum of the number of phylogenetically informative markers supporting both hypotheses ($Y_1 + Y_2$), (2) finding the corresponding line in the table in the first column, and (3) comparing the larger value to the critical value in the corresponding column. When the number of markers is greater than the critical value, H_0 must be rejected, and thus H_1 can be accepted with the corresponding level of significance.

Table S2. Critical numbers of markers for testing one-directional screening results

Y_1+Y_2	critical *Y1*	α<5%	5% border	critical *Y1*	α<1%	1% border
5	5	0.0312	4.8			
6	6	0.0156	5.5			
7	7	0.0078	6.2	7	0.0078	7.1
8	7	0.0352	6.8	8	0.0039	7.8
9	8	0.0195	7.5	9	0.0020	8.5
10	9	0.0107	8.1	10	0.0010	9.2
11	9	0.0327	8.7	10	0.0059	9.9
12	10	0.0193	9.3	11	0.0032	10.5
13	10	0.0461	10.0	12	0.0017	11.2
14	11	0.0287	10.6	12	0.0065	11.9
15	12	0.0176	11.2	13	0.0037	12.5
16	12	0.0384	11.8	14	0.0021	13.2
17	13	0.0245	12.4	14	0.0064	13.8
18	13	0.0481	13.0	15	0.0038	14.4
19	14	0.0318	13.6	15	0.0096	15.1
20	15	0.0207	14.2	16	0.0059	15.7
21	15	0.0392	14.8	17	0.0036	16.3
22	16	0.0262	15.4	17	0.0084	17.0
23	16	0.0466	15.9	18	0.0053	17.6
24	17	0.0320	16.5	19	0.0033	18.2
25	18	0.0216	17.1	19	0.0073	18.8
26	18	0.0378	17.7	20	0.0047	19.4
27	19	0.0261	18.3	20	0.0096	20.0
28	19	0.0436	18.9	21	0.0063	20.7
29	20	0.0307	19.4	22	0.0041	21.3
30	20	0.0494	20.0	22	0.0081	21.9

Y_1, Y_2 are the numbers of markers supporting two different tree topologies, Y_1+Y_2 is their sum, α is the level of significance, "Critical Y_1" is the critical value of Y_1 for rejecting H_0 (see equation 16 in the Manuscript) at the level of significance α; "α% border" is the critical value of Y_1 calculated by the normal distribution approximation (see equation 27 in the Manuscript) at the level of significance α.

For example, if we have 16 markers supporting the first tree topology and 5 markers supporting the second tree topology, their sum 16+5=21 can be found in column 1 at line 21 of Table S2. Then, with a selected significance value of $\alpha<5\%$, we can reject H_0 and accept H_1 because the tested value 16 is larger than the critical value 15 shown in column 2. We cannot reject H_0 at a selected significance value of $\alpha<1\%$ because the tested value 16 is smaller than the critical value 17. Thus we can support the first tree configuration with a significance of $p<0.05$.

Testing: two- and three- directional searches

Here, we present the next two tables (Tables S3 and S4) for testing possible tree/ancestral hybridization/polytomy configurations based on a two-step criterion (see 22). Table S3 is essential for testing the ancestral hybridization hypothesis against any other possible tree/polytomy configuration.

Table S3. Critical numbers of markers for testing possible ancestral hybridization cases.

Y_2+Y_3	critical Y_2-Y_3	$\alpha<5\%$	5% border	Y_2+Y_3	critical Y_2-Y_3	$\alpha<1\%$	1% border
6	6	0.0313	5.8	6			
7	7	0.0156	6.2	7			
8	8	0.0078	6.5	8	8	0.0078	8.3
9	7	0.0391	6.9	9	9	0.0039	8.7
10	8	0.0215	7.2	10	10	0.0020	9.1
11	9	0.0117	7.5	11	11	0.0010	9.5
12	8	0.0386	7.8	12	10	0.0063	9.9
13	9	0.0225	8.1	13	11	0.0034	10.3
14	10	0.0129	8.3	14	12	0.0018	10.6
15	9	0.0352	8.6	15	11	0.0074	11.0
16	10	0.0213	8.8	16	12	0.0042	11.3
17	9	0.0490	9.1	17	13	0.0023	11.6
18	10	0.0309	9.3	18	12	0.0075	11.9
19	11	0.0192	9.5	19	13	0.0044	12.2
20	10	0.0414	9.8	20	14	0.0026	12.5
21	11	0.0266	10.0	21	13	0.0072	12.8
22	12	0.0169	10.2	22	14	0.0043	13.1
23	11	0.0347	10.4	23	15	0.0026	13.4
24	12	0.0227	10.6	24	14	0.0066	13.6
25	11	0.0433	10.8	25	15	0.0041	13.9
26	12	0.0290	11.0	26	14	0.0094	14.1
27	13	0.0192	11.2	27	15	0.0059	14.4
28	12	0.0357	11.4	28	16	0.0037	14.6
29	13	0.0241	11.6	29	15	0.0081	14.9
30	12	0.0428	11.7	30	16	0.0052	15.1

Y_2+Y_3 is the sum of the two smallest of three values (numbers of markers) from the presence/absence pattern [Y_1,Y_2,Y_3]; "Critical Y_2-Y_3" is the critical value of their difference for rejecting H_{23} (see equation 16 in the Manuscript) at the level of significance α; $\alpha<5\%$ and $\alpha<1\%$ are levels of significance for the statistical test; "α% border" is the critical value of differences calculated by the normal distribution approximation (see equation 26 in the Manuscript) at the level of significance α.

In Table S4 we present the critical values for the numbers of markers and the significance levels for supporting the bifurcating tree hypotheses, rejecting other trees, and for cases of polytomy (e.g., due to incomplete lineage sorting) for data from unbiased, two-directional searches in which ancestral hybridization has been preliminarily rejected.

Table S4. Critical numbers of markers for testing a tree hypothesis against other possible trees and polytomy

$n=Y_1+Y_2+Y_3$	critical Y_1	α< 5%	5% border	critical Y_1	α<1%	1% border
3	3	0.0370	2.8			
4	4	0.0123	3.4			
5	4	0.0453	3.9	5	0.041	4.6
6	5	0.0178	4.4	6	0.014	5.2
7	5	0.0453	4.9	6	0.069	5.7
8	6	0.0197	5.4	7	0.026	6.3
9	6	0.0424	5.8	7	0.083	6.8
10	7	0.0197	6.3	8	0.034	7.3
11	7	0.0386	6.7	8	0.088	7.8
12	8	0.0188	7.2	9	0.039	8.3
13	8	0.0347	7.6	9	0.088	8.8
14	9	0.0174	8.1	10	0.040	9.3
15	9	0.0308	8.5	10	0.085	9.7
16	9	0.0500	8.9	11	0.040	10.2
17	10	0.0273	9.4	11	0.080	10.7
18	10	0.0433	9.8	12	0.039	11.2
19	11	0.0241	10.2	12	0.074	11.6
20	11	0.0376	10.6	13	0.037	12.1
21	12	0.0212	11.1	13	0.068	12.5
22	12	0.0327	11.5	14	0.035	13.0
23	12	0.0480	11.9	14	0.062	13.4
24	13	0.0284	12.3	15	0.032	13.9
25	13	0.0415	12.7	15	0.056	14.3
26	14	0.0248	13.1	15	0.092	14.8
27	14	0.0359	13.5	16	0.050	15.2
28	15	0.0216	13.9	16	0.082	15.6
29	15	0.0311	14.3	17	0.045	16.1
30	15	0.0435	14.7	17	0.072	16.5

$n=Y_1+Y_2+Y_3$ is the number of detected phylogenetic markers in all three directions; "critical Y_1" is the closest critical value of Y_1 for rejecting hypothesis $(\bar{H}_{1+})$ (see equation 16 in the Manuscript) at significance level α; α<5% and α<1% are levels of significance for the statistical test; "α% border" is the critical value of Y_1 calculated by the normal distribution approximation (see equation 25 in the Manuscript) at the level of significance α.

The statistical evaluation of the phylogenetic pattern [Y_1,Y_2,Y_3], where $Y_1 \geq Y_2 \geq Y_3$ includes the following steps: (1) if $Y_2>Y_3$, calculate the difference Y_2-Y_3 and the sum Y_2+Y_3, (2) check the significance level of the difference Y_2-Y_3 in Table S3 by finding the corresponding number in the

first column, and (3) compare this difference to the critical values; if difference Y_2-Y_3 is smaller than the critical value, the hybridization hypothesis is rejected.

If hybridization is rejected, the full sum $n=Y_1+Y_2+Y_3$ is calculated, identified in the first column of Table S4, and Y_1 then compared with the given critical value in the corresponding row. If Y_1 is larger or equal to the critical value then the polytomy hypothesis $\bar{H}_{1+}$ can be rejected at the selected level of significance, and we have no reason to reject the tree under consideration.

For example, if we have 16 markers supporting the first tree topology, 5 markers supporting the second tree topology, and 4 markers supporting the third tree topology, the sum of their two smallest values (5+4=9) can be found in column 1 al line 21 of Supplementary Table S3. Then, at a selected significance value of α<5% we cannot reject H_0 because the tested value of the difference (5-4=1) is smaller than the critical value 7. Hence we can reject the hybridization scenario. The next step of testing is to find the sum of all markers (16+5+4=25) in column 1 of Table S4. Then, with a selected significance value of α<1%, the critical value of Y_1 (15) indicated in column 5 of the same line is smaller than our value 16 and we can reject H_0 and accept H_1. Thus we can support the first tree configuration with a significance of $p<0.05$.

Of course, if there are no conflicting markers in the presence/absence patterns [*n*:0:0] or the smallest values are equal [*n*:*k*:*k*], the first test can be omitted and the statistical evaluation can be restricted to Table S4.
