# Supplementary material for: Incomplete Lineage Sorting and Hybridization Statistics for Large-Scale Retroposon Insertion Data
Source: PLoS Comput Biol. 2016 Mar 11;12(3):e1004812. doi: 10.1371/journal.pcbi.1004812 (PMC4788455; doi:10.1371/journal.pcbi.1004812)
Supplement: S4 Appendix — Contains sections: Estimation of the confidence intervals for the drift time; evaluation of confidence interval for the drift time; simulation of confidence intervals for the drift time τ in a diploid population; examples of calculation of Ne confidence intervals for ancestral lineages. Supplementary Figure S1 Fig; Supplemantary Tables S5-S8. (PDF) [file pcbi.1004812.s004.pdf]

Confidence intervals for the drift time τ

Method for estimating the confidence intervals for the drift time

According to our proposed mathematical model (equation S1.38 - S1.39 in S1 Appendix) we have:

$$p_1 = 1 - \frac{2}{3}\Psi(\tau_1)$$
$$p_2 = p_3 = \frac{1}{3}\Psi(\tau_1) \tag{S4.1}$$

where

$$\Psi(\tau) = \frac{e^{-\tau}}{1 + \frac{n_0}{n_1}\left(\tau + e^{-\tau} - 1\right)}\ , \tag{S4.2}$$

$\tau_1 = \dfrac{T_1}{2N_1}$ is the drift time for the branch AB,

N_1 the average effective population size of the first branch before the split (at the period $[t_0, t_1]$);

n_1 the average number of new insertions of retroelements per generation on the first branch (at the period $[t_0, t_1]$), and

n_0 the average number of new insertions of retroelements per one generation in an ancestral population. If hypothesis H_1 is accepted and there are no reasons to reject the *C-tree* hypothesis (Fig. 2a), it is possible to construct a confidence interval for the parameter p_1. Specifying a confidence probability $\beta = 1 - \alpha$, the lower and upper bounds of the confidence interval (p_1', p_1'') are determined by the conditions:

$$I_{p_1'}(Y_1, Y_2 + Y_3 + 1) = \frac{\alpha}{2} \tag{S4.3}$$

$$I_{p_1''}(Y_1 + 1, Y_2 + Y_3) = 1 - \frac{\alpha}{2}, \tag{S4.4}$$

here Y_1, Y_2, Y_3 are values of η_1, η_2, η_3 obtained from the retrophylogenomic reconstruction. Values of p_1' can be obtained by using tables of incomplete beta functions, or calculated by the formula:

$$p_1' = \frac{Y_1}{Y_1 + (Y_2 + Y_3 + 1)\cdot F\left(2(Y_2 + Y_3 + 1), 2Y_1, 1 - \frac{\alpha}{2}\right)}, \tag{S4.5}$$

$$p_1'' = \frac{(Y_1 + 1)\cdot F\left(2(Y_1 + 1), 2(Y_2 + Y_3), 1 - \frac{\alpha}{2}\right)}{(Y_1 + 1)\cdot F\left(2(Y_1 + 1), 2(Y_2 + Y_3), 1 - \frac{\alpha}{2}\right) + Y_2 + Y_3}\ , \tag{S4.6}$$

where $F(d_1, d_2, P)$ is the P-quantile for the F-distribution (Fisher-Snedecor distribution), determined from the condition:

$$P(F(d_1, d_2) < F(d_1, d_2, P)) = P\ , \tag{S4.7}$$

and $F(d_1, d_2)$ is a random variable having an F-distribution with parameters d_1 and d_2. Based on the value p_1' and p_1'' we can construct lower and upper bounds for $\tau_1 = \frac{T_1}{2N_1}$, by substituting them instead of p_1 into the first of equations (S4.1) and solving the resulting equation. Note that if $Y_1 \gg Y_2 + Y_3$ (in particular: $Y_2 + Y_3 = 0$), it is appropriate to consider the confidence region: $p_1 > p_1'$ (and $\tau_1 > \tau_1'$ - respectively). Then, in the formulas (S4.3) and (S4.5) $\frac{\alpha}{2}$ must be replaced with α.

Evaluation of confidence interval for the drift time

As shown above, if there are no reasons to reject the *C-tree* hypothesis (Fig. 2a), based on the value p_1' we can construct confidence intervals for $\tau_1 = \frac{T_1}{2N_1}$. The Tables (Tables S4-S6) show the results of the confidence intervals for p_1 and the corresponding bounds for the relative duration of branch AB normalized to effective population sizes (τ_1 - drift-time) (with values of the ratio $\frac{n_1}{n_0}$ = 0.5, 1, and 2, where this ratio indirectly characterizes the proportion that the population size of branch AB is of the ancestral populations). The confidence interval indicates the range of possible values of drift time for the branch AB. In cases when more than three lineages are studied, this information can provide additional information on the structure of the joint species tree. Information of the effective population size and generation time on branch AB can provide some estimation of branch lengths.

As an example of constructing confidence intervals of drift time we can apply the data presented in Waddell et al. (Waddell, Kishino, and Ota 2001), given the following SINE marker presence/absence pattern [50:8:6] for Human-Mouse, Human-Cow, and Mouse-Cow, respectively. Considering β=0.95 (α=0.05), we can use formulas (S4.5)-(S4.6). According to the F-distribution tables, we have: F(30; 100; 0.975) = 1.715, F(102; 28; 0.975) = 1.920, thus:

$$p_1' = \frac{50}{50 + 15 \cdot 1.715} = 0.660\ ,$$

$$p_1'' = \frac{51 \cdot 1.920}{51 \cdot 1.920 + 8 + 6} = 0.875\ .$$

Substituting these values instead of p_1 in the equation:

$$p_1 = 1 - \frac{2}{3}\Psi(\tau_1) \qquad \text{(S4.8)}$$

we solve the equations obtained for $r = \frac{n_1}{n_0}$ ratio values of 0.5, 1, and 2. Figure S1 illustrates the construction of graphs of the corresponding functions. The result is a 95% confidence interval for τ_1: (0.602; 1.395) at r = 0.5; (0.553; 1.246) at r = 1; and (0.489; 1.071) at r = 2. Note that the left ends of the obtained intervals can be regarded as the lower bounds of the unilateral 97.5% confidence intervals. The lower bounds of the unilateral 95% confidence intervals can be found by substituting in (S4.5) the value of F(30, 100, 0.95) = 1.573. Then:

$$p_1' = \frac{50}{50 + 15 \cdot 1.573} = 0.679\,.$$

Hence, solving the equation (S4.8), we obtain the lower confidence limits for τ_1: 0.650 at r = 0.5; 0.595 at r = 1; 0.523 at r = 2. These confidence intervals are in good agreement with the results of the point estimation (0.863) of Waddell et al. (Waddell, Kishino, and Ota 2001), which is located in the middle of the calculated confidence interval at r=1. However, the confidence intervals provide stronger estimations of drift time and indicate not only lower and higher probable values of this parameter, but also the variability range of this interval.

Simulation of confidence intervals for the drift time τ in a diploid population.

Because the drift time or branch length τ is one of the main parameters of our statistical model and can be directly calculated for inserted phylogenetic marker distributions, we performed a simple Monte-Carlo simulation according to Waxman (Waxman, 2011) of the different branch lengths, assuming a fixed effective population size N_e. In our simulation of a sexually reproducing diploid population of N_e=100, in each generation, 100 phylogenetic markers were randomly inserted into the population. Each new marker was introduced randomly in an individual of the population, implementing a new allelic variation. We define the loss of a marker if we could not find this marker in the current population, and define fixation of a marker if this marker is present homogeneously in all members of the population. After $2 \cdot N_e = 200$ generations, we split the first progenitor population into two, with an initial N_e=100. Next, after T generations (where T is a changing variable ranging between $50 \le T \le 500$ stepwise by 50), we split one of the new populations into two as described above. After the second split we stopped introducing new markers and waited until all previously introduced markers were lost or fixed in all three populations. For each value of T we performed 100 repetitions. Than we calculated

$\tau = \frac{T}{2 \cdot N_e}$ for each value of T, and collected information about markers shared between two of the three final populations in all possible combinations. Based on marker distributions among three populations, 95% confidence interval limits of tau were calculated as described above. The results are presented in Table S7. These results are strongly correlated to the simulation presets, e.g., medians of the confidence intervals are slightly different to simulation parameters (maximum of difference is 5.2%). Interestingly the significance of the branch for the drift time value 0.25 is already strongly supported ($p<0.0053$).

Examples of calculation of *Ne* confidence intervals for ancestral lineages

Testing a hypothetical SINE presence/absence pattern from the work of Waddell et al. (2001): (50:8:6) for human-mouse, human-cow, and mouse-cow, respectively, we obtain a 95% confidence interval for τ_1: (0.553; 1.246) at a ratio $\frac{n_0}{n_1}$ of normalized population sizes of young branches equivalent to 1 (see above). Taking into account that $\tau_{AB} = \frac{T_{AB}}{2N_{AB}}$ Waxman (2011), where T_{AB} is the length of ancestral branch between connected lineages in generations, and N_{AB} is the effective ancestral population size on the ancestral brunch, we can give confidence interval limits for the effective population size or the length of the ancestral branches. For example, using parameters from the above example of Waddell et al. (2001): 1 year per generation and the length of the branch equal to 5 million years, we can estimate confidence intervals for N_e. For 95% significance, the confidence interval of N_e will be between 2.01×10^6 and 4.52×10^6, which agrees with the $N_e = 2.90\times10^6$ presented by Waddell et al. (2001).

Based on the significant support for a common ancestry of Pinnipedia and Musteloidea (Doronina et al, 2015), we can estimate the confidence intervals for the drift time τ for this ancestral branch. Taking the ratio of normalized effective population sizes $\frac{n_0}{n_1} = 1$, then the 95% confidence intervals for the drift time τ is between 0.313 and 0.534. The length of the common arctoid ancestral branch is estimated at 2.1 million years (Eizirik et al, 2010). Based on the expectation that ancestral arctoids were of relatively small size (Flynn & Nedbal 1998, Wang et al. 2005), we can assume a generation time of about 5 years. With these parameters a rough estimation of the ancestral effective population size is possible. Within a 95% confidence interval, the estimated N_e is 0.39×10^6- 0.67×10^6. We should mention that this calculation is based on a rough estimation of the generation time, which is difficult to verify after more than 40 million years of evolution. In addition only short internal phylogenetic branches with conflicting

patterns of presence/absence markers provide sufficient information for such estimation. In any event the calculations lead only to a very weak and unstable indication because retroposon markers accumulate randomly over time and their frequencies depend on the activity of master genes whose activity can differ between different phylogenetic episodes.

Supplementary References

Waddell, P. J., H. Kishino, and R. Ota. 2001. A phylogenetic foundation for comparative mammalian genomics. Genome Inform **12**:141-153.

Waxman, D. 2011. A unified treatment of the probability of fixation when population size and the strength of selection change over time. Genetics **188**: 907-913.

Eizirik E, Murphy WJ, Koepfli KP, Johnson WE, Dragoo JW, et al. (2010) Pattern and timing of diversification of the mammalian order Carnivora inferred from multiple nuclear gene sequences. Mol Phylogenet Evol 56: 49-63.

Flynn JJ, Nedbal MA (1998) Phylogeny of the Carnivora (Mammalia): congruence vs incompatibility among multiple data sets. Mol Phylogenet Evol 9: 414-426.

Wang XM, McKenna MC, Dashzeveg D (2005) Amphicticeps and Amphicynodon (Arctoidea, Carnivora) from Hsanda Gol Formation, central Mongolia and phylogeny of basal arctoids with comments on zoogeography. Am Mus Nov 3483: 1–57.

Figure S1

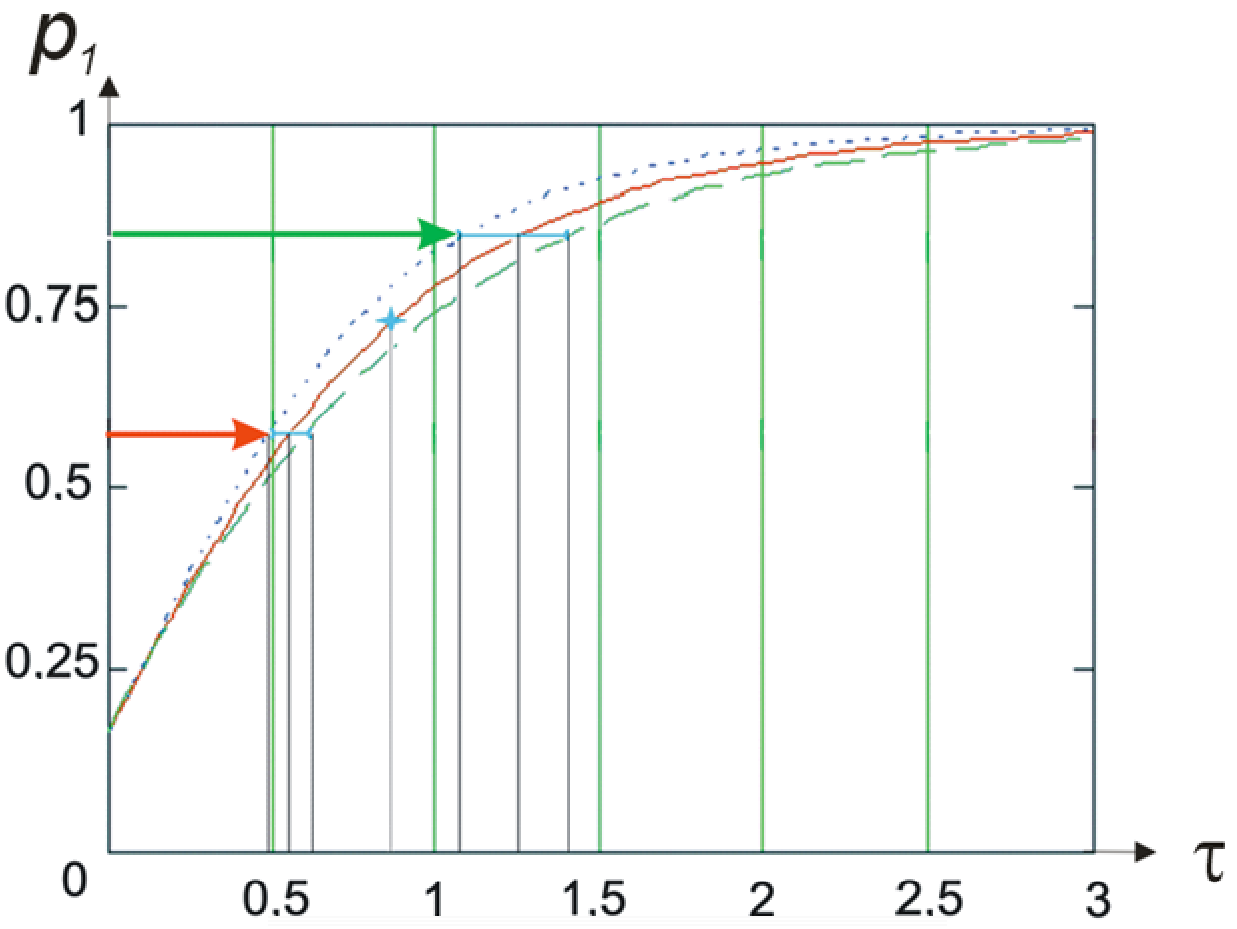

Figure S1. Finding 95% confidence limits for drift time.

The graph shows curves for the function $p_1 = 1 - \frac{2}{3} \cdot \frac{e^{-\tau_1}}{1 + \frac{n_0}{n_1}\left(\tau + e^{-\tau_1} - 1\right)}$ calculated for different ratios of $\frac{n_1}{n_0}$ (green dashed line at 0.5; red line at 1, blue dotted line at 2). Green and red arrows show upper and lower 95% confidential interval bounds, respectively. Light blue lines with brackets indicate differences in values, dependent on the $\frac{n_1}{n_0}$ ratio. Light blue star marks the value of drift time 0.863, discussed in Waddell et al. (Waddell, Kishino, and Ota 2001). Black vertical lines project values to horizontal axis.

Table S5. Lower bounds of 95% and 99% confidence intervals of diffusion time for branch AB in the "*C-tree*".

Lower bound of 95% confidential interval						Lower bound of 99% confidential interval					
n	Y_1	p_1	τ_1			n	Y_1	p_1	τ_1		
			r=0.5	r=1	r=2				r=0.5	r=1	r=2
3	3	0.368	0.053	0.053	0.051	-	-	-	-	-	-
4	4	0.473	0.223	0.214	0.199	-	-	-	-	-	-
5	5	0.549	0.363	0.341	0.309	5	5	0.398	0.100	0.098	0.094
5	4	0.343	0.014	0.014	0.014	-	-	-	-	-	-
6	6	0.607	0.480	0.446	0.398	6	6	0.464	0.208	0.200	0.186
6	5	0.418	0.132	0.128	0.122	-	-	-	-	-	-
7	7	0.652	0.582	0.536	0.473	7	7	0.518	0.304	0.287	0.263
7	6	0.479	0.234	0.224	0.208	7	6	0.357	0.035	0.035	0.034
7	5	0.341	0.012	0.012	0.012	-	-	-	-	-	-
8	8	0.688	0.671	0.614	0.539	8	8	0.562	0.388	0.364	0.328
8	7	0.529	0.325	0.306	0.279	8	7	0.410	0.119	0.116	0.111
8	6	0.400	0.103	0.101	0.097	-	-	-	-	-	-
9	9	0.717	0.751	0.684	0.598	9	9	0.599	0.464	0.432	0.386
9	8	0.571	0.405	0.379	0.341	9	8	0.456	0.194	0.187	0.175
9	7	0.450	0.185	0.178	0.167	9	7	0.344	0.016	0.016	0.015
9	6	0.345	0.017	0.017	0.017	-	-	-	-	-	-
10	10	0.741	0.823	0.747	0.650	10	10	0.631	0.533	0.493	0.438
10	9	0.606	0.478	0.444	0.396	10	9	0.496	0.263	0.251	0.231
10	8	0.493	0.259	0.246	0.227	10	8	0.388	0.084	0.083	0.080
10	7	0.393	0.092	0.090	0.087	-	-	-	-	-	-
11	11	0.762	0.889	0.804	0.698	11	11	0.658	0.596	0.548	0.484
11	10	0.636	0.544	0.502	0.445	11	10	0.530	0.326	0.308	0.281
11	9	0.530	0.326	0.307	0.280	11	9	0.428	0.147	0.143	0.135
11	8	0.436	0.160	0.155	0.146	11	8	0.340	0.009	0.009	0.009
11	7	0.350	0.025	0.025	0.024	-	-	-	-	-	-
12	12	0.779	0.949	0.857	0.742	12	12	0.681	0.655	0.599	0.527
12	11	0.661	0.605	0.556	0.490	12	11	0.560	0.384	0.360	0.325
12	10	0.562	0.387	0.363	0.328	12	10	0.463	0.206	0.198	0.184
12	9	0.473	0.223	0.213	0.198	12	9	0.378	0.068	0.067	0.065
12	8	0.391	0.088	0.087	0.083	-	-	-	-	-	-
13	13	0.794	1.005	0.905	0.783	13	13	0.702	0.708	0.647	0.567
13	12	0.684	0.661	0.605	0.532	13	12	0.587	0.438	0.409	0.367
13	11	0.590	0.444	0.414	0.371	13	11	0.494	0.260	0.248	0.228
13	10	0.505	0.281	0.266	0.245	13	10	0.412	0.122	0.119	0.114
13	9	0.427	0.147	0.142	0.135	13	9	0.339	0.009	0.009	0.009
13	8	0.355	0.032	0.032	0.032	-	-	-	-	-	-
14	14	0.807	1.057	0.951	0.821	14	14	0.720	0.759	0.691	0.603
14	13	0.703	0.713	0.650	0.570	14	13	0.611	0.489	0.453	0.404
14	12	0.615	0.497	0.461	0.410	14	12	0.522	0.311	0.294	0.268
14	11	0.534	0.334	0.315	0.287	14	11	0.443	0.173	0.167	0.157
14	10	0.460	0.201	0.193	0.180	14	10	0.373	0.060	0.059	0.057
14	9	0.390	0.088	0.086	0.083	-	-	-	-	-	-
15	15	0.819	1.106	0.993	0.857	15	15	0.736	0.806	0.732	0.638
15	14	0.721	0.761	0.693	0.605	15	14	0.632	0.536	0.495	0.439
15	13	0.637	0.546	0.504	0.447	15	13	0.547	0.358	0.337	0.305
15	12	0.560	0.384	0.360	0.325	15	12	0.471	0.221	0.212	0.197
15	11	0.489	0.252	0.240	0.222	15	11	0.403	0.108	0.105	0.101
15	10	0.423	0.139	0.135	0.128	15	10	0.340	0.010	0.010	0.010
15	9	0.360	0.040	0.039	0.039	-	-	-	-	-	-

Notes: For the "*C-tree*" [Y_1, Y_2, Y_3], where $Y_1 \geq Y_2 \geq Y_3$: $n=Y_1+Y_2+Y_3$; Y_1 is the number of markers supporting the "*C-tree*"; p_1 is the relative number of markers supporting the AB branch in the full

set of available markers; $\tau_1 = \frac{T_1}{2N_1}$ is the drift time for the branch AB (where T_1 is the length of the branch AB in generations and N_1 is the average effective population size on the branch AB).

Table S6. Lower and higher bounds of 95% confidence intervals of diffusion time for branch AB in the "*C-tree*"

n	Y_1	p_1'	p_1''	*r*=0.5		*r*=1		*r*=2	
				τ_1'	τ_1''	τ_1'	τ_1''	τ_1'	τ_1''
6	5	0,359	0,996	0,039	4,121	0,038	3,739	0,038	3,322
7	6	0,421	0,996	0,137	4,250	0,133	3,862	0,126	3,437
8	7	0,473	0,997	0,224	4,363	0,215	3,968	0,199	3,538
8	6	0,349	0,968	0,024	2,467	0,024	2,203	0,023	1,906
9	8	0,518	0,997	0,303	4,462	0,287	4,063	0,262	3,628
9	7	0,400	0,972	0,103	2,565	0,100	2,292	0,096	1,987
10	9	0,555	0,997	0,374	4,551	0,351	4,148	0,317	3,708
10	8	0,444	0,975	0,174	2,653	0,168	2,373	0,158	2,059
10	7	0,348	0,933	0,021	1,884	0,021	1,678	0,021	1,443
11	10	0,587	0,998	0,438	4,632	0,409	4,225	0,367	3,781
11	9	0,482	0,977	0,240	2,733	0,229	2,445	0,212	2,125
11	8	0,390	0,940	0,087	1,965	0,086	1,750	0,083	1,506
12	11	0,615	0,998	0,498	4,706	0,462	4,295	0,411	3,849
12	10	0,516	0,979	0,300	2,805	0,284	2,512	0,260	2,185
12	9	0,428	0,945	0,148	2,038	0,144	1,815	0,136	1,563
12	8	0,349	0,901	0,023	1,575	0,023	1,404	0,023	1,206
13	12	0,640	0,998	0,553	4,774	0,510	4,360	0,452	3,911
13	11	0,546	0,981	0,355	2,872	0,334	2,573	0,303	2,240
13	10	0,462	0,950	0,204	2,104	0,196	1,875	0,183	1,616
13	9	0,386	0,909	0,080	1,643	0,079	1,464	0,076	1,258
14	13	0,661	0,998	0,605	4,837	0,556	4,421	0,490	3,968
14	12	0,572	0,982	0,407	2,934	0,381	2,630	0,343	2,292
14	11	0,492	0,953	0,257	2,166	0,245	1,931	0,226	1,665
14	10	0,419	0,916	0,133	1,706	0,129	1,520	0,123	1,306
14	9	0,351	0,872	0,027	1,379	0,027	1,232	0,027	1,059
15	14	0,681	0,998	0,653	4,896	0,598	4,477	0,526	4,022
15	13	0,595	0,983	0,456	2,991	0,424	2,683	0,379	2,340
15	12	0,519	0,957	0,306	2,224	0,289	1,982	0,264	1,711
15	11	0,449	0,922	0,183	1,764	0,176	1,571	0,165	1,350
15	10	0,384	0,882	0,077	1,438	0,076	1,284	0,073	1,103

For the *C-tree* [Y_1, Y_2, Y_3], where $Y_1 \geq Y_2 \geq Y_3$: $n=Y_1+Y_2+Y_3$; Y_1 is the number of markers supporting the *C-tree*; p_1 is the relative number of markers supporting the AB branch in the full set of available markers; $\tau_1 = \frac{T_1}{2N_1}$ is the drift time for the branch AB (where T_1 is the length of the branch AB in generations and N_1 is the average effective population size at the branch AB); (') and ('') denote lower and higher bounds of confidence intervals, respectively, for the corresponding values.

Table S7. Lower and higher bounds of 99% confidence intervals of diffusion time for branch AB in the "*C-tree*"

n	Y_1	p_1'	p_1''	*r*=0.5		*r*=1		*r*=2	
				τ_1'	τ_1''	τ_1'	τ_1''	τ_1'	τ_1''
8	7	0,368	0,999	0,053	5,752	0,053	5,301	0,052	4,813
9	8	0,415	0,999	0,127	5,855	0,123	5,401	0,118	4,909
10	9	0,456	0,999	0,194	5,947	0,187	5,490	0,174	4,995
10	8	0,352	0,989	0,028	3,336	0,028	3,002	0,027	2,635
11	10	0,491	1,000	0,256	6,031	0,244	5,570	0,225	5,074
11	9	0,392	0,990	0,089	3,418	0,088	3,079	0,084	2,705
12	11	0,523	1,000	0,313	6,107	0,296	5,644	0,270	5,145
12	10	0,427	0,991	0,146	3,493	0,142	3,148	0,134	2,769
12	9	0,345	0,970	0,017	2,506	0,017	2,238	0,017	1,938
13	12	0,551	1,000	0,366	6,177	0,344	5,712	0,311	5,211
13	11	0,459	0,992	0,200	3,561	0,192	3,212	0,179	2,829
13	10	0,379	0,972	0,070	2,574	0,069	2,301	0,067	1,994
14	13	0,576	1,000	0,415	6,242	0,388	5,774	0,349	5,272
14	12	0,488	0,992	0,249	3,625	0,238	3,272	0,220	2,884
14	11	0,411	0,974	0,120	2,638	0,117	2,358	0,112	2,046
14	10	0,342	0,947	0,013	2,071	0,013	1,845	0,013	1,590
15	14	0,598	1,000	0,462	6,303	0,430	5,833	0,384	5,330
15	13	0,514	0,993	0,296	3,684	0,280	3,327	0,257	2,935
15	12	0,439	0,976	0,167	2,697	0,161	2,412	0,152	2,095
15	11	0,373	0,951	0,060	2,130	0,059	1,898	0,058	1,637

For the *C-tree* [Y_1, Y_2, Y_3], where $Y_1 \geq Y_2 \geq Y_3$: $n=Y_1+Y_2+Y_3$; Y_1 is the number of markers supporting the *C-tree*; p_1 is the relative number of markers supporting the AB branch in the full set of available markers; $\tau_1 = \frac{T_1}{2N_1}$ is the drift time for the branch AB (where T_1 is the length of the branch AB in generations and N_1 is the average effective population size at the branch AB); ′ and ″ denote lower and higher bounds of confidential intervals, respectively, for the corresponding values.

Table S8. Results of simulation of diffusion time 95% confidential intervals.

branch length (T)	drift time (τ)	95% confidential limits of τ		calculated median τ	significance of the tree (KKSC test)
		lower bound	upper bound		
50	0.25	0.050±0.003	0.446±0.005	0.248±0.004	$0.52\cdot10^{-2}$
100	0.50	0.251±0.007	0.752±0.005	0.501±0.006	$0.56\cdot10^{-5}$
150	0.75	0.439±0.006	1.054±0.007	0.747±0.006	$0.41\cdot10^{-9}$
200	1.00	0.632±0.005	1.393±0.006	1.013±0.005	$0.44\cdot10^{-14}$
250	1.25	0.790±0.006	1.704±0.006	1.247±0.006	$0.66\cdot10^{-18}$
300	1.50	0.962±0.007	2.142±0.005	1.552±0.006	$0.54\cdot10^{-21}$
350	1.75	1.073±0.009	2.410±0.007	1.742±0.008	$0.45\cdot10^{-24}$
400	2.00	1.311±0.010	2.682±0.009	1.996±0.010	$0.14\cdot10^{-37}$
450	2.25	1.549±0.012	2.950±0.011	2.250±0.011	$0.59\cdot10^{-53}$
500	2.50	1.788±0.011	3.215±0.013	2.503±0.012	$0.81\cdot10^{-69}$

"Branch length (T)" and "drift time (τ)" were directly set in the simulation process, other parameters were calculated based on the simulation outcome (average from 100 simulations for each value are presented in the table).
