# Supplementary material for: Incomplete Lineage Sorting and Hybridization Statistics for Large-Scale Retroposon Insertion Data
Source: PLoS Comput Biol. 2016 Mar 11;12(3):e1004812. doi: 10.1371/journal.pcbi.1004812 (PMC4788455; doi:10.1371/journal.pcbi.1004812)
Supplement: S5 Appendix — Supplementary Table S9. (PDF) [file pcbi.1004812.s005.pdf]

Supplementary Table S9: Comparative presentation of strategies to evaluate the reliability of relationships based on presence/absence data.

targets	markers	phylogeny	KKSC tree (P)	Waddell (P)	PAUP_Dollo (bootstrap)	MrBayes (bootstrap)	Chi-square (P)
**Lagomorpha**	3:0:0	rabbit<br>+hare<br>-red rock hare<br>-pika	0.0370*	0.0370*	100*	62	0.0497*<br>(0.2528)
**Landbirds**	2:0:0	zebra finch<br>+gulls<br>-stork<br>-chicken	0.1111	0.1111	100*	-	0.1353<br>(0.5698)
**Marsupials**	1:0:0	marsupial mole<br>+bandicoot<br>-kangaroo<br>-monito del monte	0.3333	0.3333	100*	-	0.3679<br>(0.8825)
**Rodentia**	8:2:0	mouse<br>+guinea pig<br>-squirrel<br>(-rabbit)	0.0034*	na	97.5*	82	0.0055*<br>(0.0199*)
**Carnivora**	192:74:60	walrus<br>+ferret<br>-polar bear<br>-dog	3e-21*	na	100*	100*	1e-36*
**Musteloidea**	3:1:0	raccoon<br>+ferret<br>-red panda<br>-walrus	0.1111	0.0617	84	-	0.1737<br>(0.4577)
**Placentalia**	9:8:5	human<br>+elephant<br>-armadillo<br>-opossum	0.2930	na	56	-	0.5537
**Wu 1991**	27:13:0	example 1	2e-4* (hyb.)<br>0.0385* (tree)	na	100*	99*	1.15e-6*
**Waddell 2010**	50:8:6	example 2	2e-13*	na	100*	100*	1e-38

The KKSC test was applied corresponding to the presented new calculations (http://retrogenomics.uni-muenster.de:3838/KKSC_significance_test/). The cumulative P values of Waddell were taken from Waddell et al. 2001 (their Table 3). PAUP was used to reconstruct split support values (bootstrap) using the version PAUP*4.0b10 and the irrev.up option of character transformation. MrBayes v3.2.5 was used to calculate Bayesian support values for branch points using the standard discrete model (binary) and ctype irreversible. Finally, a classical Chi-square test was applied to calculate P-values. For small numbers, we performed the Yates correction (P-value in brackets). All examples are described in in the main body of the text. Lagomorpha 3:0:0 describes three different hypotheses favoring a close relationship between Orytolagus+Lepus to the exclusion of Pronolagus and Ochotona. Examples 1 and 2 are conceived cases to demonstrate the KKSC statistics in special relationships (e.g., under hybridization). Asterisks denote significant splits.

References:

Swofford DL. 2002. PAUP*: phylogenetic analysis using parsimony (*and other methods. 4.0b10 ed. Sunderland (MA): Sinauer Associates, Inc.

Ronquist F, Teslenko M, van der Mark P, Ayres DL, Darling A, Höhna S, Larget B, Liu L, Suchard MA, Huelsenbeck JP 2012. MrBayes 3.2: efficient Bayesian phylogenetic inference and model choice across a large model space. Syst Biol. 61:539–542.
